# Estimating individual subjective values of emotion regulation strategies

**DOI:** 10.1038/s41598-023-40034-7

**Published:** 2023-08-15

**Authors:** Christoph Scheffel, Josephine Zerna, Anne Gärtner, Denise Dörfel, Alexander Strobel

**Affiliations:** 1 Chair of Differential and Personality Psychology, Faculty of Psychology, Technische Universität Dresden, 01069 Dresden, Germany; 2 Center for Information Services and High Performance Computing, Technische Universität Dresden, 01069 Dresden, Germany

**Keywords:** Human behaviour, Emotion, Decision, Personality

## Abstract

**Abstract:**

Individuals have a repertoire of emotion regulation (ER) strategies at their disposal, which they can use more or less flexibly. In ER flexibility research, strategies that facilitate goal achievement are considered adaptive and therefore are subjectively valuable. Individuals are motivated to reduce their emotional arousal effectively and to avoid cognitive effort. Perceived costs of ER strategies in the form of effort, however, are highly subjective. Subjective values (SVs) should therefore represent a trade-off between effectiveness and subjectively required cognitive effort. However, SVs of ER strategies have not been determined so far. We present a new paradigm for quantifying individual SVs of ER strategies by offering monetary values for ER strategies in an iterative process. *N* = 120 participants first conducted an ER paradigm with the strategies distraction, distancing, and suppression. Afterwards, individual SVs were determined using the new CAD paradigm. SVs significantly predicted later choice for an ER strategy (χ^2^ (4, *n* = 119) = 115.40, *p* < 0.001, BF_10_ = 1.62 × 10^21^). Further, SVs were associated with Corrugator activity (*t* (5, 618.96) = 2.09, *p* = 0.037, *f*^2^ = 0.001), subjective effort (*t* (5, 618.96) = − 13.98, *p* < 0.001, *f*^2^ = 0.035), and self-reported utility (*t* (5, 618.96) = 29.49, *p* < 0.001, *f*^2^ = 0.155). SVs were further associated with self-control (*t* (97.97) = 2.04, *p* = 0.044, *f*^2^ = 0.002), but not with flexible ER. With our paradigm, we were able to determine subjective values. The trait character of the values will be discussed.

**Protocol registration:**

The stage 1 protocol for this Registered Report was accepted in principle on July 19, 2022. The protocol, as accepted by the journal, can be found at: 10.17605/OSF.IO/FN9BT.

## Introduction

The ability to modify emotional experiences, expressions, and physiological reactions^1^ to regulate emotions is an important cognitive skill. It is therefore not surprising that emotion regulation (ER) has substantial implications for well-being and adaptive functioning^2^. Different strategies can be used to regulate emotions, namely situation selection, situation modification, attentional deployment, cognitive change, and response modification^1^, and, following the taxonomy of Powers and LaBar^3^, individuals can implement ER strategies by means of different tactics. So called antecedent-focused strategies, e.g., attentional deployment and cognitive change, take effect early in the emotion generation process^1^. In contrast, response modification takes place late in the process and is therefore conceptualized as a response-focused strategy^1^. This postulated temporal sequence of ER strategies influences their effectiveness. Albeit it is meta-analytically proven that all mentioned strategies reduce subjective emotional experience, distraction as a tactic of attentional deployment and (expressive) suppression as a tactic of response modulation showed only small to medium effect sizes (distraction: *d*_*+*_ = 0.27; suppression: *d*_*+*_ = 0.27). In contrast, distancing as tactic of cognitive change showed the highest effectiveness with an effect size of *d*_*+*_ = 0.45^4^.

Psychophysiological measures provide further important information on the effectiveness of emotion regulation strategies (for an overview, see Zaehringer et al.^5^). Compared to cardiovascular, electrodermal, and pupillometric autonomic responses, facial electromyography has been reported consistently across studies to be influenced by emotion regulation with even medium effect sizes. For example, studies have shown that reappraisal of negative emotion is associated with reduced activity of the corrugator supercilii (associated with anger, sadness, and fear) with *d* = − 0.32^5^. In addition, the levator labii superioris (associated with disgust) has also been associated with reduced activity during reappraisal^6^. Similar effects have been reported for suppression^6^, distancing^7^, and distraction^8^. Importantly, results on electromyographic measures seem to be more consistent compared to other autonomic measures, likely because they are specific to emotional valence and its changes.

Similarly to the differences in short term effectiveness, these tactics from three different strategies are also related to different medium and long-term consequences. In particular, strategies that do not change the emotional content of the situation, for instance by taking a neutral perspective (i.e., distraction and suppression) are presumed to be disadvantageous in the longer term. Thus, the self-reported habitual use of suppression is associated with more negative affect and lower general well-being^9^. In addition, a number of ER strategies, e.g., rumination and suppression, have been associated with mental disorders (for meta-analytic review, see Aldao et al.^10^), which led to the postulation of *adaptive* (such as reappraisal, acceptance) and *maladaptive* (such as suppression, rumination) ER strategies. For example, it was shown that maladaptive ER strategies (rumination and suppression) mediate the effect between neuroticism and depressive symptoms^11^.

The postulation of *adaptive* and *maladaptive* ER strategies has been challenged by the concepts of ER repertoire and ER flexibility. Within this framework, *maladaptive* refers to inflexible ER strategy use or use of strategies that are hindering goal achievement^12^. Adaptive flexible ER requires a large repertoire of ER strategies^12^. The term “repertoire” can be defined as the ability to utilize a wide range of regulatory strategies in divergent contextual demands and opportunities^13^. A growing number of studies report findings about the repertoire of emotion regulation strategies and its relationship to psychopathology^14–16^. Additionally, greater ER flexibility is related to reduced negative affect and therefore beneficial in daily life^17^.

How do people choose strategies from their repertoire? Similarly to the expectancy-value model of emotion regulation^18^ it could be assumed, that people also assign a value to an ER strategy reflecting the usefulness of this strategy for goal achieving. Evidence from other psychological domains (e.g., intertemporal choice^19^) shows that subjective values (SVs) are attributed to the choice options on the basis of which the decision is made. Research on ER choice has identified numerous factors that influence the choice of ER strategies, which can be seen as indirect evidence for factors influencing SVs^20^. For example, a study found that the intensity of a stimulus or situation plays a role in the choice^21^. Higher intensity of the (negative) stimulus lead to a choice of rather disengaging tactics of attentional deployment, like distraction^20,21^. ER choice was further influenced by, among others, extrinsic motivation (e.g., monetary incentives), motivational determinants (i.e., hedonic regulatory goals), and effort^20,22^. Nonetheless, there are only few studies to date that examined the required effort of several strategies in more detail and compared them with each other. Furthermore, the research on ER choice lacks information regarding the strategies that were *not* chosen in each case. It is unclear whether people had clear preferences or whether the choice options were similarly attractive.

We assume that people choose the strategy that has the highest value for them at the moment. The value is determined against the background of goal achievement in the specific situation: A strategy is highly valued if it facilitates goal achievement^12^. One certainly central goal is the regulation of negative affect. The effectiveness of ER strategies should therefore influence the respective SV. A second, intrinsic, and less obvious goal is the avoidance of effort^23^. When given the choice, most individuals prefer tasks that are less effortful^24^. Cognitive effort avoidance has been reported in many contexts, for example in affective context^25^, the context of decision making^26^, and executive functions^27^, and is associated with Need for Cognition (NFC)^28^, a stable measure of the individual pursuit and enjoyment of cognitive effort^29,30^. In the area of emotion regulation, too, there are initial indications that people show a tendency towards effort avoidance. Across two studies, we could show in previous work that the choice for an ER strategy is mainly influenced by the effort required to implement a given strategy^22^. In our studies, participants used the strategies distancing and suppression while inspecting emotional pictures. Afterwards, they choose which strategy they wanted to use again. Participants tended to re-apply the strategy that was subjectively less effortful, even though it was subjectively not the most effective one - in this case: suppression. Moreover, the majority of participants stated afterwards the main reason for their choice was effort. We assume therefore that, although individuals trade off both factors - effectiveness and effort - against each other, effort should be the more important predictor for SVs of ER strategies. In addition, perceived utility should have an impact on SVs. A strategy that is less effortful and can objectively regulate arousal (i.e., is effective), but is not subjectively perceived as useful, should have a low SV. SVs of ER Strategies could therefore be helpful to describe the ER repertoire^12^ more comprehensively. Depending on the flexibility of a person, different patterns of SVs could be conceivable: A person with high flexibility would show relatively high SVs for a number of strategies. This would mean that all strategies are a good option for goal achievement. A second person with less flexibility, however, would show high SVs only for one strategy or low SVs for all of the strategies. This in turn would mean that there is only a limited amount of strategies in the repertoire to choose from. Subsequently, the ability to choose an appropriate strategy for a specific situation is also limited.

So far we have not seen any attempt in ER choice research to determine individual SVs of ER strategies. However, this would be useful to describe interindividual differences in the preference of ER strategies and the ER repertoire more comprehensively. To investigate this question, the individual SVs of each strategy available for selection would have to be determined. Promising approaches can be found in studies on difficulty levels of effortful cognitive tasks.

Individual SVs of effortful cognitive tasks have been quantified using the Cognitive Effort Discounting Paradigm (COG-ED)^29^.

**Figure 1:**
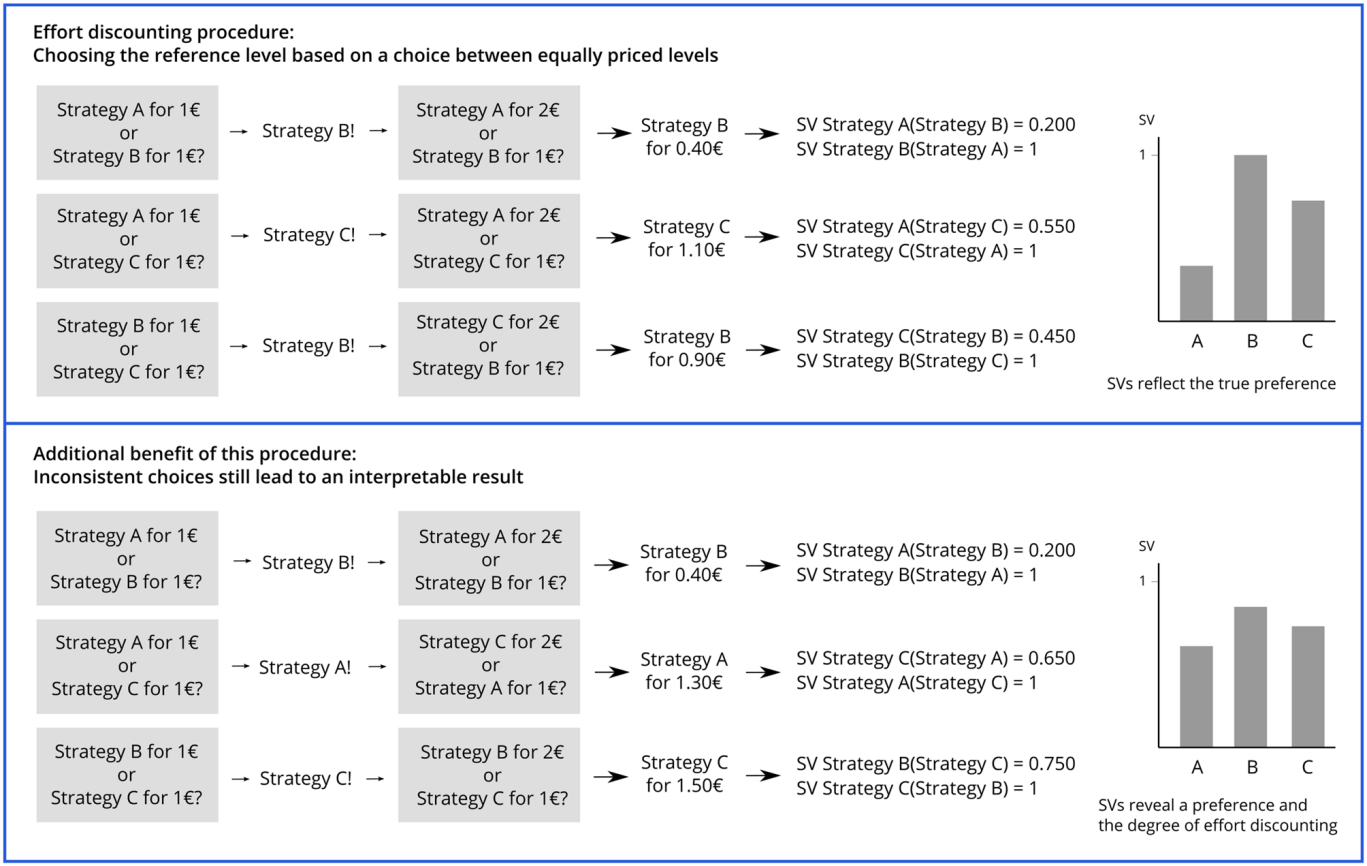
Exemplary visualization of two response patterns. In the top half, the person has a clear preference for one of the three strategies. In the lower half, they have no clear preference and therefore show an inconsistent response pattern. This pattern can be represented by our paradigm. Figure available at https://osf.io/vnj8x/, under a CC-BY4.0 license.

In the original study by Westbrook et al.^29^, cognitive load was varied using the *n*-back task, a working memory task that requires fast and accurate responses to sequentially presented stimuli. Participants had to decide in an iterative procedure whether they wanted to repeat a higher *n*-back level for a larger, fixed monetary reward, or a lower level for a smaller, varying reward, with the implicit assumption that the objectively easiest *n*-back level has the highest SV. In the present study, we want to use this paradigm to determine SVs of ER strategies. In doing so, we need to make an important change: We have to adapt the assumption that the easiest *n*-back level has the highest SV. As we have shown in previous studies, there are large inter-individual differences in the preference and perceived subjective effort of ER strategies^22^. Moreover, there is nothing like an objectively easiest ER strategy. It could be assumed, that the antecedent-focused strategies, i.e. attentional deployment and cognitive change, require less effort, because according to Gross^1^ these strategies apply when the emotional reaction has not fully developed, yet. In contrast, suppression would need ongoing effort, because it takes effect late in the emotion generating process and does not alter the emotion itself. A similar assumption has been made by Mesmer-Magnus et al.^31^, who state that Surface Acting (the equivalent to expressive suppression in emotional labor research) is supposed to continuously require high levels of energy (hence effort). Deep Acting (which refers to reappraisal), in turn, only initially needs the use of energy. This would be in conflict with findings in our previous studies, that showed that many people choose expressive suppression because they evaluated it as less effortful, hence easy^22^. Others define emotion regulation on a continuum from explicit, conscious, and effortful to implicit, unconscious, automatic and effortless^32^. This would mean, that all explicit strategies that have been proposed by the process model of emotion regulation are similarly effortful^1^. Similarly, the flexibility approach of emotion regulation also states, that there is no “best” strategy^33^. An emotion regulation attempt is adaptive, when the intended, individual goal is reached. Those attempts could also consist of sequences of regulatory efforts using different strategies, which might be effective and effortless only in this specific context. Therefore, we have to add an additional step, which precedes the other steps and where the ER option with the higher subjective value is determined. In this step, the same monetary value (i.e., 1 €) is assigned to both options. The assumption is that participants now choose the option that has the higher SV for them. In the next step we return to the original paradigm. The higher monetary value (i.e., 2 €) is assigned to the option that was not chosen in the first step and therefore is assumed to have the lower SV. In the following steps, the lower value is changed in every iteration according to Westbrook et al.^29^ until the indifference point is reached. This procedure will be repeated until all strategies have been compared. The SV of each strategy is calculated as the mean of this strategy’s SV from all comparisons. In case a participant has a clear preference for one strategy, the SV of this strategy will be 1. But our paradigm can also account for the case that a person does not have a clear preference. Then no SV will be 1, but still, the SVs of all strategies can be interpreted as absolute values and in relation to the other strategy’s SVs (see Fig. 1). In a separate study, we will test our adapted paradigm together with a *n*-back task and explore whether this paradigm can describe individuals that do not prefer the easiest *n*-back option (see Zerna, Scheffel et al.^34^).

The aim of the present study is to evaluate whether this paradigm is suitable for determining SVs of ER strategies. As a manipulation check, we first want to investigate whether the valence of the pictures is affecting subjective and physiological responding, resulting in lower subjective arousal ratings after and lower EMG activity during neutral compared to negative pictures. Second, we want to check whether the ER strategies distraction, distancing, and suppression effectively reduce subjective arousal and physiological responding compared to the active viewing condition. Third, we want to see whether the strategies subjectively require more cognitive effort than the active viewing condition, and whether participants re-apply the for them least effortful strategy. Furthermore, we want to investigate whether subjective effort, arousal ratings, subjective utility, and EMG activity predict individual subjective values of ER strategies. And lastly, we want to check whether the SV of a strategy is associated with its likelihood of being chosen again, and whether SVs reflect participants’ self-reported ER flexibility. All hypotheses are detailed in the Design Table. Exploratorily, we want to investigate whether individual SVs are related to personality traits and how individual SVs of ER strategies relate to SVs of other tasks with different demand levels, namely *n*-back.

## Method

We report how we determined our sample size, all data exclusions (if any), all manipulations, and all measures in the study^35^. The paradigm was written and presented using *PsychoPy*^36^. We used *R* with *R Studio*^37,38^ with the main packages *afex*^39^ and *BayesFactor*^40^ for all analyses. The R Markdown file used to analyze the data and write this document, as well as the raw data and the materials are freely available at https://github.com/ChScheffel/CAD. A complete list of all measures assessed in the study can be found at OSF (https://osf.io/vnj8x/) and GitHub (https://github.com/ChScheffel/CAD).

### Ethics information

The study protocol complies with all relevant ethical regulations and was approved by the ethics committee of the Technische Universität Dresden (reference number EK50012022). Prior to testing, written informed consent was obtained. Participants received 24 € in total or course credit for participation.

### Pilot data

The newly developed ER paradigm was tested in a pilot study with $N=16$ participants (9 female; age: $M = 24.1\text { }\pm \text { }SD = 3.6$). Regarding self-reported arousal, results showed significant higher subjective arousal for active viewing of negative compared to neutral pictures. However, ER strategies did not lead to a reduction of subjective arousal compared to active viewing of negative pictures. Regarding physiological responses, ER strategies were associated with reduced facial muscle activity of the *corrugator* and *levator* compared to active viewing of negative pictures. In accordance with our previous study^22^, we found that the use of ER strategies compared to active viewing was associated with increased subjective effort. All results are detailed in the OSF repository (https://osf.io/vnj8x/).

### Design

Young healthy participants (aged 18 to 30 years) were recruited using the software *ORSEE*^41^ at the Technische Universität Dresden. Participants were excluded from participation if they do not fluently speak German, had current or a history of psychological disorders or neurological trauma, or reported to take medication. Participants were invited to complete an online survey containing different questionnaires to assess broad and narrow personality traits and measures of well-being. The study consisted of two lab sessions, which took place in a shielded cabin with constant lighting. Before each session, participants received information about the respective experimental procedure and provided informed consent. In the first session participants filled out a demographic questionnaire and completed an *n*-back task with the levels one to four. Then, they completed an effort discounting (ED) procedure regarding the *n*-back levels on screen, followed by a random repetition of one *n*-back level^34^. The second session took place exactly one week after session one. Participants provided informed consent and received written instructions on the ER paradigm and ER strategies that they should apply. A brief training ensured that all participants were able to implement the ER strategies. Next, electrodes to measure facial EMG were attached and the ER task was conducted, followed by an ED procedure regarding the ER strategies. After that, participants chose one ER strategy to repeat one more time. Study data were collected and managed using REDCap electronic data capture tools hosted at Technische Universität Dresden^42,43^.

#### Psychometric measures

The online survey contained a number of questionnaires. In the focus of the current project was the Flexible Emotion Regulation Scale (FlexER)^44^.

It assesses flexible use of ER strategies with items such as “If I want to feel less negative emotions, I have several strategies to achieve this.”, which we define as ER flexibility. The items were rated on a 4-point scale ranging from “strongly agree” to “strongly disagree”.

Further psychological constructs were assessed but had no clear hypotheses in the present work and are therefore investigated only exploratory: General psychological well-being was assessed using the German version of the WHO-5 scale^45,46^. To measure resilience, the German version 10-item-form of the Connor-Davidson resilience Scale (CD-RISC)^47–49^ was used. Habitual use of ER was assessed using the German version of the Emotion Regulation Questionnaire (ERQ)^9,50^. Implicit theories of willpower in emotion control was assessed using the implicit theories questionnaire from Bernecker and Job^51^. To assess Need for Cognition, the German version short form of the Need for Cognition Scale^28,52^ was used. To assess self-control^53^, sum scores of the German versions of the following questionnaires were used: the Self-Regulation Scale (SRS)^54^, the Brief Self-Control Scale (BSCS)^55,56^, and the Barratt Impulsiveness Scale (BIS-11)^57,58^. Attentional control were assessed using the Attentional Control Scale (ACS)^59^. For more detailed information on psychometric properties of the questionnaires, please see the supplementary material.

#### Emotion regulation paradigm

The ER paradigm consisted of three parts that will be described in the following

**Figure 2:**
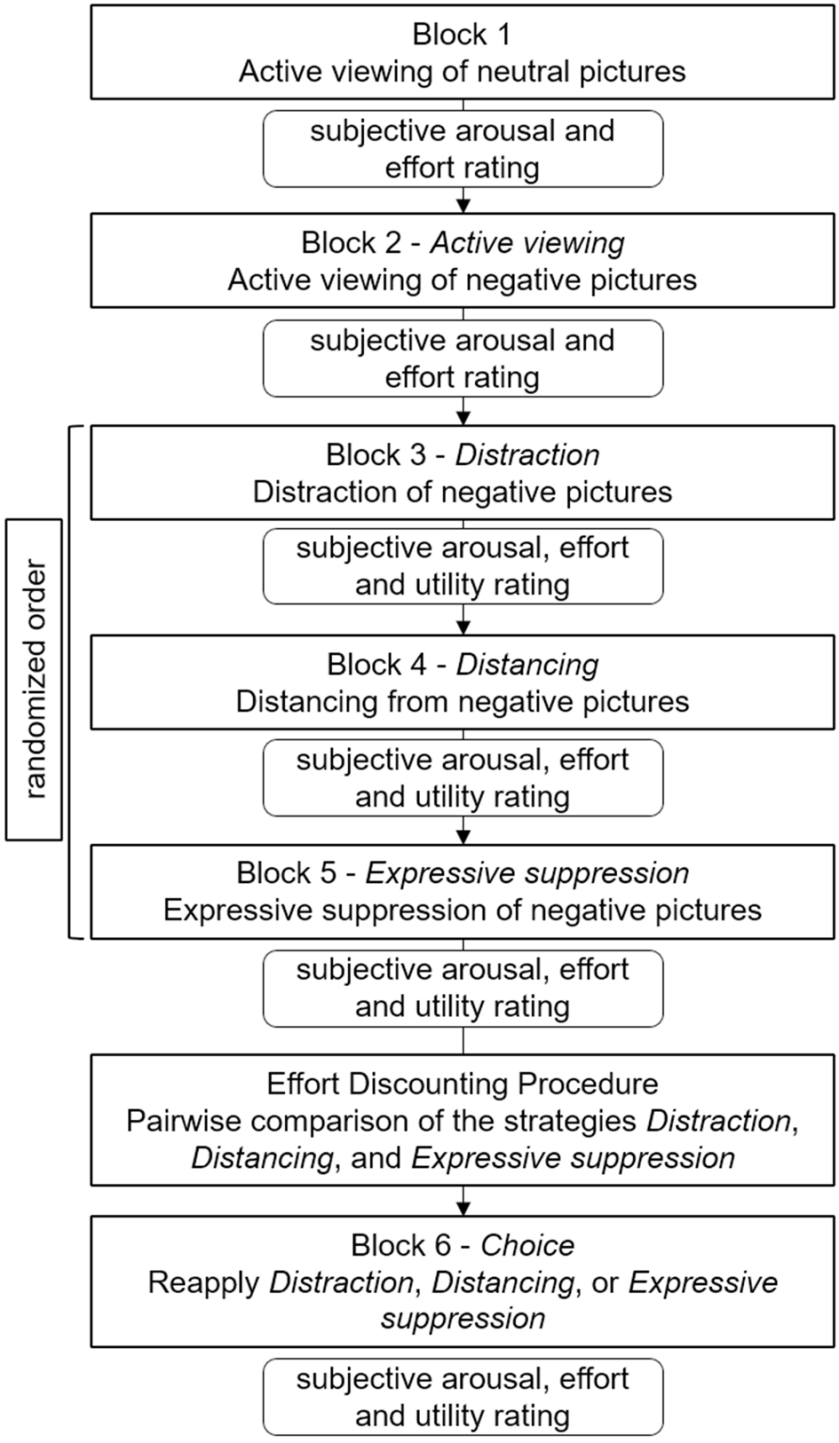
Block design of the paradigm. Every participant starts with two “active viewing” blocks containing neutral (Block 1) and negative (Block 2) pictures. Order of the regulation blocks (Blocks 3, 4, and 5) was randomized between participants. After, the discounting procedure took place. All three regulation strategies were compared pairwise. Before the last block, participants could decide which regulation strategy they wanted to reapply. Subjective arousal and effort ratings were assessed after each block using a slider on screen with a continuous scale. Figure available at https://osf.io/vnj8x/, under a CC-BY4.0 license.

- *Part one: ER task* Part one was a standard ER task in a block design (see Fig. 2), similar to paradigms previously used by our group^22^. Participants were told to actively view neutral and negative pictures (see 2.3.3) or to regulate all upcoming emotions by means of distraction, distancing, and expressive suppression, respectively. Every participant first had the condition “active viewing-neutral” that served as a baseline condition. During this block, 20 neutral pictures were presented. Participants were asked to “actively view all pictures and permit all emotions that may arise.” In the second block, participants actively viewed negative pictures. During the third, fourth, and fifth block, participants saw negative pictures and were asked to regulate their emotions using distraction, distancing, and suppression. In order to achieve distraction, participants were asked to think of a geometric object or an everyday activity, like brushing their teeth. During distancing, participants were asked to “take the position of a non-involved observer, thinking about the picture in a neutral way.” Participants were told not to re-interpret the situation or attaching a different meaning to the situation. During suppression, participants were told to “suppress their emotional facial expression.” They should imagine being observed by a third person that should not be able to tell by looking at the facial expression whether the person is looking at an emotional picture. Participants were instructed not to suppress their thoughts or change their facial expression to the opposite^22^. All participants received written instruction and completed a training session. After the training session, participants were asked about their applied ER strategies to avoid misapplication. The order of the three regulation blocks (distraction, distancing, and suppression) were randomized between participants. Each of the blocks consisted of 20 trials showing neutral (Block 1) and negative (Blocks 2, 3, 4, 5) pictures. Each trial began with a fixation cross that lasted 3 to 5 seconds (random uniform distributed). It was followed by neutral or negative pictures for a total of 6 seconds. After each block, participants retrospectively rated their subjective emotional arousal (“not at all aroused” to “very highly aroused”), their subjective effort (“not very exhausting” to “very exhausting”), and - after the regulation blocks - the utility of the respective strategy (“not useful at all” to “very useful”) on a continuous scale using a slider on screen.
- *Part two: ER effort discounting* In the second part, ER effort discounting took place. The procedure of the discounting will follow the COG-ED paradigm by Westbrook et al.^29^ with a major change. We used the following adaption that allowed the computation of SVs for different strategies without presuming that all individuals would inherently evaluate the same strategy as the easiest one: For each possible pairing (distraction vs. distancing, distraction vs. suppression, and distancing vs. suppression), each of the two strategies were presented with a monetary reward. Because there is no strategy that is objectively more difficult, we added initial comparisons asking the participants to choose between “1€ for strategy A or 1€ for strategy B”. They decided by clicking the on-screen button of the respective option. Each of the three strategy pairs were presented three times in total, in a randomized order and randomly assigned which strategy appeared on the left or right side of the screen. For each pair, the strategy that was chosen at least two out of three times was assigned the flexible starting value of 1€, the other strategy was assigned the fixed value of 2€. After this, comparisons between strategies followed the original COG-ED paradigm^29^. Each pairing was presented six consecutive times, and with each decision the reward of the strategy with the starting value of 1€ was either lowered (if this strategy was chosen) or raised (if the strategy with the fixed 2€ reward was chosen). The adjustment started at 0.50€ and each was half the adjustment of the previous step, rounded to two digits after the decimal point. If a participant always chose the strategy with the fixed 2€ reward, the other strategy’s last value on display was 1.97€, if they always choose the lower strategy, its last value was 0.03€. The sixth adjustment of 0.02€ was done during data analysis, based on the participants’ decision in the last display of the pairing. Participants were instructed to decide as realistically as possible by imagining that the monetary reward was actually available for choice.
- *Part three: ER choice* After the discounting part, participants chose which one of the three ER strategies (distraction, distancing or suppression) they wanted to re-apply. Importantly, there was no further instruction on what basis they should make their decision. Participants should make their decision freely, according to criteria they consider important for themselves. However, participants were asked to state the reasons for the decision afterwards in RedCap using a free text field. As soon as they have decided, they saw the respective instruction and the block with another 20 negative pictures started.

#### Stimuli

Pictures that were used in the paradigm were selected from the Emotional Picture Set (EmoPicS)^60^ and the International Affective Picture System (IAPS)^61^. The 20 neutral pictures (Valence (V): *M* ± *SD* = 4.81 ± 0.51; Arousal (A): *M* ± *SD* = 3.00 ± 0.65) depicted content related to the categories persons, objects, and scenes. Further, 100 negative pictures, featuring categories animals, body, disaster, disgust, injury, suffering, violence, and weapons, were used. An evolutionary algorithm^62^ was used to cluster these pictures into five sets with comparable valence and arousal values (set one: V: *M* ± *SD* = 2.84 ± 0.57, A: *M* ± *SD* = 5.62 ± 0.34; set two: V: *M* ± *SD* = 2.64 ± 0.46, A: *M* ± *SD* = 5.58 ± 0.35; set three: V: *M* ± *SD* = 2.82 ± 0.62, A: *M* ± *SD* = 5.60 ± 0.39; set four: V: *M* ± *SD* = 2.65 ± 0.75, A: *M* ± *SD* = 5.61 ± 0.41; set five: V: *M* ± *SD* = 2.74 ± 0.70, A: *M* ± *SD* = 5.63 ± 0.37). A complete list of all pictures and their classification into sets can be found in supplementary material Table S1. The five sets of negative pictures were assigned randomly to the blocks.

#### Facial electromyography

Bipolar facial electromyography (EMG) were measured for *corrugator supercilii* and *levator labii* as indices of affective valence^63^, similar to previous work by our group^7^. Two passive surface Ag/AgCl electrodes (8 mm inner diameter, 10 mm distance between electrodes) were placed over each left muscle according to the guidelines of Fridlund and Cacioppo^64^. The ground electrode was placed over the left *Mastoid*. Before electrode placement, the skin was abraded with Every abrasive paste, cleaned with alcohol, and filled with Lectron III electrolyte gel. Raw signals were amplified by a BrainAmp amplifier (Brain Products Inc., Gilching, Germany). Impedance level were kept below 10 $k\Omega$. Data were sampled at 1000 Hz, filtered, rectified and integrated. A 20 Hz high pass (order 8), a 300 Hz low pass (order 8), and a 50 Hz notch filter was applied to both signals. *Corrugator* and *levator* EMG was analyzed during the 6 s of picture presentation. EMG data were baseline-corrected using a time window of 2 s prior to stimulus onset^63^. Last, the sampling rate was changed to 100 Hz, and EMG data were averaged for each condition and each participant.

### Sampling plan

Sample size calculation was done using G*Power^65,66^. In a meta-analysis of Zaehringer and colleagues^5^, effect sizes of ER on peripheral-physiological measures were reported: To find an effect of $d=-0.32$ of ER on *corrugator* muscle activity with $\alpha =.05$ and $\beta =.95$, data of at least $N=85$ have to be analyzed. Power analyses of all other hypotheses yielded smaller sample sizes. However, if participants withdraw from study participation, technical failures occur, or experimenter considers the participant for not suitable for study participation (e.g., because the participant does not follow instructions or shows great fatigue), respective data will also be excluded from further analyses. Therefore, we aimed to collect data of $N=120$ participants, about $50$ more data sets, than necessary. Detailed information on power calculation for each hypothesis can be found in the design table.

### Analysis plan

Data collection and analysis were not performed blind to the conditions of the experiments. Data of whole participants were excluded from analysis if participants withdraw their consent or they stated that they did not follow experimental instructions. EMG data of subjects were excluded from analysis if errors occured during recording. No further data exclusions were planned. The level of significance was set to $\alpha =.05$. For hypotheses H1-4, repeated measures analysis of variance (rmANOVA) were conducted and estimated marginal means were computed using the *afex* package^39^. Greenhouse-Geisser-corrected degrees of freedom and associated $p$-values were reported when the assumption of sphericity was violated. If the within-subjects factor of interest was significant, pairwise contrasts were calculated using Bonferroni adjustment for multiple testing. Proportion of explained variance $\eta _{p}^{2}$ was reported as a measure of effect size.

- *Effect of valence on arousal and facial EMG* To examine the impact of valence of emotional pictures on subjective arousal ratings (H1a), a rmANOVA with the factor valence (neutral and negative) for the strategy active viewing was conducted. To examine the impact of valence on physiolocigal responding (H1b and H1c), a rmANOVA with the factor valence (neutral and negative) for the strategy active viewing was conducted for EMG *corrugator* and *levator* activity.
- *Effects of emotion regulation on arousal, facial EMG, and effort* To investigate the effects of the three ER strategies on subjective arousal (H2a), another rmANOVA with the factor strategy (active viewing - negative, distraction, distancing, and suppression) for subjective arousal ratings was conducted. To examine the effects of the three ER strategies on physiological responding (H3a and H3b), another rmANOVA with the factor strategy (active viewing-negative, distraction, distancing, and suppression) for EMG *corrugator* and *levator* activity was conducted. To examine the effect of ER strategies on subjective effort (H4a), a rmANOVA with the factor strategy (active viewing - negative, distraction, distancing, and suppression) for subjective effort ratings was conducted.
- *Subjective values of emotion regulation strategies* For each ER strategy, SVs were calculated as follows: first, the SV of the flexible strategy was set to 1, because that strategy was preferred when equal rewards were offered. Second, to obtain the SV of the fixed strategy (the minimum relative reward required for participants to choose the flexible strategy over the fixed strategy), the value 0.02€ was added to or subtracted from the last monetary value of the flexible strategy, depending on the participant’s last choice. The resulting value of the flexible strategy was divided by 2€. This yielded an SV of the fixed strategy between 0 and 1, with values closer to 0 indicating a stronger aversion to the fixed strategy compared to the flexible strategy. The final SV per strategy for each participant was computed by averaging the SVs of each strategy across pairings.

To explore the association between subjective effort (H5a), subjective arousal (H5b), subjective utility (H5c), and physiological responding (H5d,e) on SVs, a multilevel model (MLM) was specified using the *lmerTest* package^67^. First, ER strategies were recoded and centered for each subject according to their individual SVs: The strategy with the highest SV was coded as -1, the strategy with the second highest SV 0, and the strategy with the lowest SV was coded as 1. Restricted maximum likelihood (REML) was applied to fit the model. A random slopes model of SVs including subjective effort (effort ratings), subjective arousal (arousal ratings), utility (utility ratings), and physiological responses (*corrugator* and *levator* activity) as level-1-predictors was specified.

$$\begin{aligned} \begin{aligned}{}&SV \sim strategy\ + \text {effort rating} + \text {arousal rating} + \text {utility rating} + corrugator \text { activity} \\&\quad + levator \text { activity} + (strategy|subject) \end{aligned} \end{aligned}$$

Level-1-predictors were centered within cluster^68^. Residuals of the final model were inspected visually. Intraclass correlation coefficient (ICC), $\rho$, was reported for each model (null model, as well as full model). The presented MLM followed the conceptualization of Zerna, Scheffel, et al.^34^

To investigate whether individual SVs predict ER choice (H7a), a $\chi {2}$ test with predicted choice (highest SV of each participant) and actual choice was computed. Furthermore, an ordinal logistic regression with the dependent variable choice and independent variables SVs of each strategy was computed.

The association between flexible ER and SVs of ER strategies (H7b) was investigated with a linear regression using the individual *intercept* and *slope* of each participants’ SVs to predict their FlexER score. To this end, for each participant, SVs were sorted by magnitude in descending order and entered as dependent variable in a linear model, with strategy (centered, i.e., − 1, 0, 1) as independent variable. The resulting *intercept* informs about the extent to which an individual considers any or all of the ER strategies as useful for regulation their emotion, while the *slope* informs about the flexibility in the use of emotion regulation strategies. The individual intercepts and slopes were entered as predictors in a regression model with the FlexER score as dependent variable. A positive association with the predictor *intercept* would indicate that overall higher SVs attached to ER strategies predicts higher scores on the FlexER scale. A positive association with the predictor *slope* would indicate that less negative slopes, i.e., a smaller preference for a given ER strategy, would be associated with a higher score of the FlexER scale.

The influence of personality traits on SVs were investigated exploratorily. Therefore, the MLM specified above was extended by the level-2-predictors NFC and self-control.

For each result of the analyses, both $p$-values and Bayes factors BF_10_, calculated using the *BayesFactor* package^40^, were reported. Bayes factors were calculated using the default prior widths of the functions *anovaBF*, *lmBF* and *regressionBF*.

## Results

### Participants and descriptive statistics

Data collection took place between the 16th of August 2022 and the 3rd of February 2023. A total of $N=151$ participants completed the online survey and were invited to participate in the two lab sessions. The first session was attended by $N=124$ participants^34^, and $N=121$ participants also completed the second session. We excluded the data of $n=1$ person from the present analyses because they stated that they did not follow the instructions. Therefore, the final sample consisted of $N=120$ participants (100 female; age: $M\text { }\pm \text { }SD =22.5\text { }\pm \,3.0$ years old), which is 1.4 times more than what the highest sample size calculation required. Please note that the sample size for a few analyses may be smaller due to failure of EMG recording ($n=1$) and failure to record utility ratings ($n=18$).

### Confirmatory analyses

#### Manipulation checks

##### Effect of valence on arousal and facial EMG

To explore whether negative pictures evoked emotional arousal and physiological responding, we conducted separate rmANOVAs for the active viewing condition with the predictors subjective arousal, *corrugator* and *levator* activity. Descriptive values of each predictor per condition can be found in Table 1. We found a significant main effect of valence on subjective arousal ($F(1, 119) = 399.95$, $p < .001$, $\hat{\eta }^2_G = .589$, 90% CI $[.498, .659]$, $\textrm{BF}_{\text {10}} = 2.76 \times 10^{48}$), *corrugator* activity ($F(1, 117) = 27.73$, $p < .001$, $\hat{\eta }^2_G = .111$, 90% CI $[.037, .206]$, $\textrm{BF}_{\text {10}} = 8.05 \times 10^{18}$), and *levator* activity ($F(1, 117) = 8.87$, $p = .004$, $\hat{\eta }^2_G = .039$, 90% CI $[.002, .111]$, $\textrm{BF}_{\text {10}} = 251.32$). Post-hoc contrasts indicated that negative pictures successfully increased emotional arousal and physiological responding (please see Tables S.4 to S.6 and Figs. S.1 to S.3 in the supplementary material).

##### Effect of emotion regulation on arousal and facial EMG

To investigate whether ER strategies reduced emotional arousal and physiological responding, we conducted separate rmANOVAs comparing the four instructed strategies (active viewing, distraction, distancing, suppression) with respect to subjective arousal, *corrugator* and *levator* activity. We found a significant main effect of strategy on subjective arousal ($F(2.71, 322.55) = 7.39$, $p < .001$, $\hat{\eta }^2_G = .015$, 90% CI $[.000, .036]$, $\textrm{BF}_{\text {10}} = 157.74$), *corrugator* activity ($F(1.76, 206.02) = 13.70$, $p < .001$, $\hat{\eta }^2_G = .056$, 90% CI $[.019, .094]$, $\textrm{BF}_{\text {10}} = 1.96 \times 10^{10}$), and *levator* activity ($F(1.54, 180.41) = 19.95$, $p < .001$, $\hat{\eta }^2_G = .089$, 90% CI $[.043, .134]$, $\textrm{BF}_{\text {10}} = 7.82 \times 10^{18}$), indicating that regulation strategies reduced subjective arousal and physiological responding. For detailed information on post-hoc contrasts, please see Tables S.7 to S.9 and Figs. S.4 to S.6 in the supplementary material.

**Table 1:** $M \pm SD$ of subjective arousal, subjetive effort, subjective utility, corrugator activity, and levator activity for each condition.

	Subjective arousal	Subjective effort	Subjective utility	Corrugator activity (in mV)	Levator activity (in mV)
$View_{neu}$	26.6 ± 39.1	18.1 ± 27.4		0.04 ± 6.99	0.09 ± 1.84
$View_{neg}$	187.8 ± 87.3	49.4 ± 62.3		1.03 ± 7.21	0.58 ± 3.2
Distraction	158.1 ± 92.5	208.5 ± 96.1	216.6 ± 93.2	0 ± 7.67	-0.05 ± 1.16
Distancing	164 ± 87.2	189.8 ± 92.3	214.8 ± 78.6	0.25 ± 1.92	0.01 ± 1
Suppression	168.6 ± 95.8	158.3 ± 99.5	229.3 ± 95	0.07 ± 3.78	-0.03 ± 0.92

##### Effect of emotion regulation of effort

**Figure 3:**
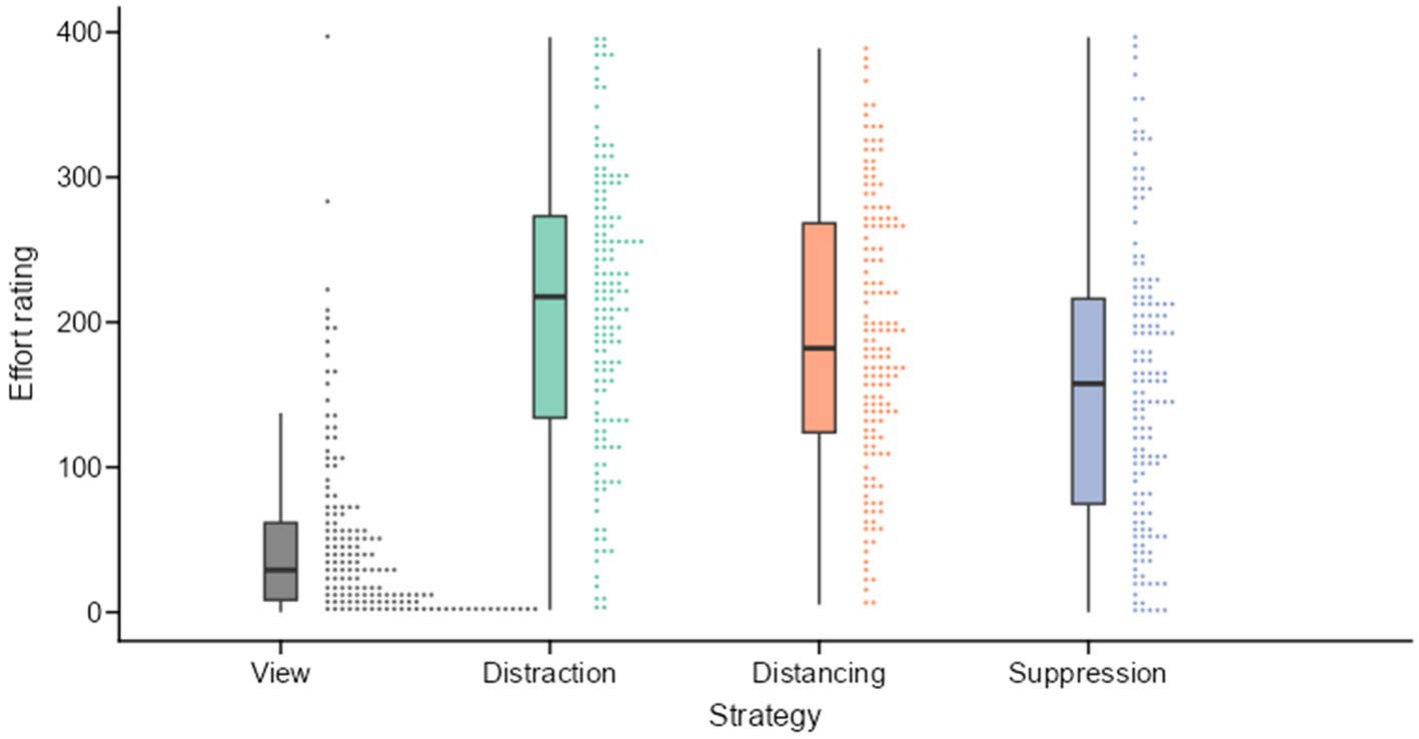
Subjective effort ratings visualized as boxplots. Dots represent individual effort ratings placed in 150 quantiles. Figure available at https://osf.io/vnj8x/, under a CC-BY-4.0 license.

To investigate whether ER strategies required cognitive effort, we conducted an rmANOVA comparing the subjective effort ratings of four strategies (active viewing, distraction, distancing, suppression). We found a significant main effect of strategy ($F(2.92, 347.65) = 128.47$, $p < .001$, $\hat{\eta }^2_G = .327$, 90% CI $[.261, .384]$, $\textrm{BF}_{\text {10}} = 1.77 \times 10^{53}$; see Fig. 3). Post-hoc contrasts showed significantly higher subjective effort for distraction ($t(357) = -17.92$, $p_\mathrm {Tukey(4)} < .001$, $\textrm{BF}_{\text {10}} = 3.61 \times 10^{30}$), distancing ($t(357) = -15.82$, $p_\mathrm {Tukey(4)} < .001$, $\textrm{BF}_{\text {10}} = 1.60 \times 10^{28}$), and suppression ($t(357) = -12.26$, $p_\mathrm {Tukey(4)} < .001$, $\textrm{BF}_{\text {10}} = 1.27 \times 10^{19}$) compared to active viewing. Moreover, we found significantly lower effort during suppression compared with distraction ($t(357) = 5.66$, $p_\mathrm {Tukey(4)} < .001$, $\textrm{BF}_{\text {10}} = 1.61 \times 10^{6}$) and distancing ($t(357) = 3.55$, $p_\mathrm {Tukey(4)} = .002$, $\textrm{BF}_{\text {10}} = 29.19$).

Cognitive effort also played the most important role in the subsequent choice of strategy, which resembled previous findings of our group^22^. The majority of participants (45.40%) stated that they chose the strategy that was easiest for them to implement, 24.40% stated they chose the strategy that was most effective, and 11.80% stated their chosen strategy was the easiest *and* most effective. A more detailed list of all reasons, including those given by participants who stated none of the three options above, can be found online on OSF (https://osf.io/vnj8x/).

#### Subjective values of ER strategies and their predictors

Individual SVs could be determined for 120 participants for all three ER strategies. SVs ranged between 0.005 and 1.00. Nearly all ($n=$ 119) participants had one SV of 1.0, indicating a clear preference for one ER strategy over the other two. Absolute preferences for ER strategies were relatively equally distributed: $n=$ 41 participants assigned their highest SV to distraction, $n=$ 36 to distancing, and $n=$ 43 to suppression.

To investigate which variables can predict individual SVs of ER strategies, a multilevel model approach was chosen. The ICC of the null model was $ICC=$ 0.19, indicating that the level-2 predictor *subject* accounted for 19.10% of total variance. The preregistered model showed a correlation of $r=$ 0.95 between the random effects *subjects* and *recoded strategy* ($\textrm{BF}_{\text {10}}$ of the variable *strategy*: $\textrm{BF}_{\text {10}} = \infty$). Our model explained 90.4% of variance and thus we assumed our model was overfitted because we included *recoded strategy* as the random slope. We therefore set a new model without *recoded strategy* as the random slope factor to estimate the influence of predictors on SVs more precisely. The second model followed the specification:

$$\begin{aligned} \begin{aligned} SV \sim \text {effort rating} + \text {arousal rating} + \text {utility rating} + corrugator \text { activity} \\\ + levator \text { activity} + (1 |subject) \end{aligned} \end{aligned}$$

The second model explained 41.5% of variance. All results of the second model are in Table 2.

**Table 2:** Results of the multilevel model predicting subjective values of ER strategies.

Parameter	Beta	*SE*	*p*-value	$f^{2}$	Random effects (SD)
Intercept	$8.03 \times 10^{-1}$	0.012	$<.001$		0.114
Effort	$-6.85 \times 10^{-4}$	0.000	$<.001$	0.035	
Arousal	$-7.84 \times 10^{-5}$	0.000	0.317	0.000	
Utility	$1.42 \times 10^{-3}$	0.000	$<.001$	0.155	
Corrugator activity	$7.45 \times 10^{-3}$	0.004	0.037	0.001	
Levator activity	$5.32 \times 10^{-3}$	0.003	0.070	0.001	

The predictors effort rating ($\hat{\beta } = -0.001$, 95% CI $[-0.001, -0.001]$, $t(5,618.96) = -13.98$, $p < .001$), utility rating ($\hat{\beta } = 0.001$, 95% CI $[0.001, 0.002]$, $t(5,618.96) = 29.49$, $p < .001$), and *corrugator* activity ($\hat{\beta } = 0.007$, 95% CI $[0.000, 0.014]$, $t(5,618.96) = 2.09$, $p = .037$) showed a significant association with SVs. Beta values were relatively small, so the respective effect size $f^{2}$ was calculated as the explained variance. The predictor utility rating showed the greatest effect size of all predictors ($f^{2}=$ 0.155), indicating that utility rating explained 15.5% of variance in SVs. Effort rating showed an effect size of $f^{2}=$ 0.035. The effect sizes of all other predictors were negligibly small ($f^{2}< 0.01$).

#### Associations between subjective values and flexible ER

**Figure 4:**
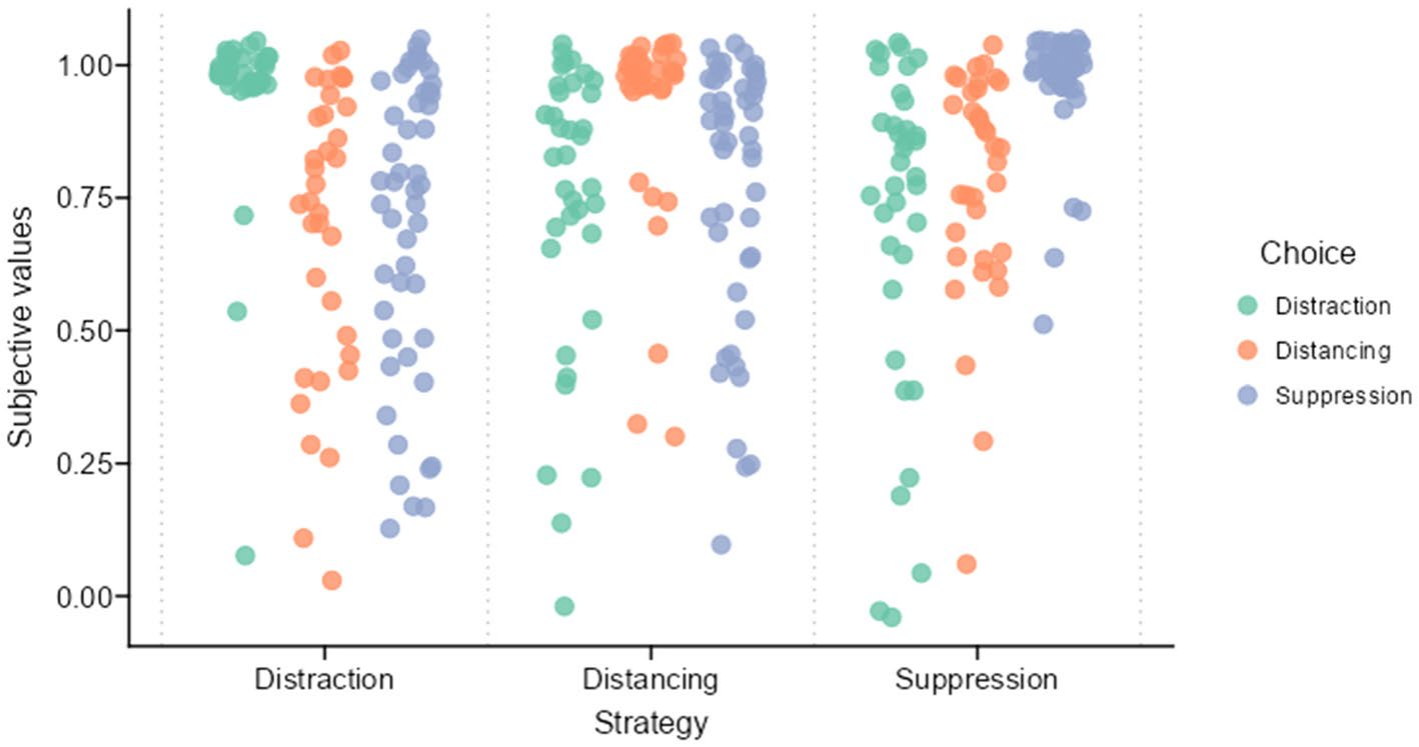
Individual subjective values per ER strategy, grouped by choice in last experimental block. Each dot indicates SV of one participant, the colours indicate their choice in last experimental block. The scatter has a horizontal jitter of 0.40 and a vertical jitter of 0.05. *N* = 120. Figure available at https://osf.io/vnj8x/, under a CC-BY-4.0 license.

To investigate the ecological validity of the calculated subjective values of ER strategies, we tested whether SVs were associated with the actual choice of participants in the last experimental block. Therefore, a $\chi ^{2}$ test with predicted choice (i.e., the strategy with the highest SV of each participant) and actual choice was computed. There was a significant association between predicted choice and actual choice ($\chi ^2(4, n = 119) = 115.40$, $p < .001$, $\textrm{BF}_{\text {10}} = 1.62 \times 10^{21}$; see Fig. 4).

We then conducted an ordinal regression with the dependent variable *choice* and the individual SVs of all three strategies as independent variables. Overall model fit was fair with $R^{2}=$ 0.27. The SV of the strategy distraction contributed significantly to the model ($b = -6.29$, 95% CI $[-10.81, -3.02]$, $z = -3.21$, $p = .001$, $\textrm{BF}_{\text {10}}=$ 2.00). The estimated odds ratio indicated a higher chance of choosing the strategy distraction when the SV of that strategy was higher. Additionally, the predictor SV of the strategy suppression contributed significantly to the model ($b = 2.70$, 95% CI $[0.83, 4.84]$, $z = 2.67$, $p = .008$, $\textrm{BF}_{\text {10}}=$ 1.99). The estimated odds ratio indicated that a participant was more likely to later choose suppression, when the SV of the strategy suppression was higher.

Lastly, we investigated whether SVs were associated with ER flexibility. We conducted a logistic regression to inspect whether participants’ individual slopes and intercepts of ordered SVs could predict their ER flexibility score. We found neither a significant association between slopes and FlexER score ($b = -0.36$, 95% CI $[-1.28, 0.56]$, $t(117) = -0.77$, $p = .444$, $\textrm{BF}_{\text {10}} = 0.72$), nor between intercepts and FlexER score ($b = 1.32$, 95% CI $[-1.38, 4.02]$, $t(117) = 0.97$, $p = .336$, $\textrm{BF}_{\text {10}} = 0.85$). However, model fit was relatively low ($R^2 = .03$, $F(2, 117) = 1.93$, $p = .150$).

### Exploratory analyses

Because associations between self-control, the investment trait Need for Cognition (NFC), and both effort discounting and demand avoidance have been reported^29,34,69^, we wanted to investigate the influence of self-control and NFC on individual SVs of ER strategies. The starting point for this was the adapted MLM reported above (Table 2). Only predictors that had previously shown a significant association with SVs were included in the model together with the level-2 predictors self-control and NFC. The third model followed the specification:

$$\begin{aligned} \begin{aligned}{}&SV \sim \text {effort rating} + \text {utility rating} + corrugator \text { activity} \\&\quad + \text {self-control} + \text {NFC} + (1 |subject) \end{aligned} \end{aligned}$$

The predictor effort rating ($\hat{\beta } = -0.001$, 95% CI $[-0.001, -0.001]$, $t(5,620.93) = -14.26$, $p < .001$) showed a negative association with SVs, while utility rating ($\hat{\beta } = 0.001$, 95% CI $[0.001, 0.002]$, $t(5,620.93) = 33.28$, $p < .001$) and *corrugator* activity ($\hat{\beta } = 0.008$, 95% CI $[0.001, 0.015]$, $t(5,620.93) = 2.12$, $p = .034$) showed a positive association with SVs. In addition, a positive association was also found between self-control and SVs ($\hat{\beta } = 0.024$, 95% CI $[0.001, 0.048]$, $t(97.97) = 2.04$, $p = .044$). However, the effect size of this effect was negligibly small ($f^{2}=$ 0.002). Detailed information can be found in Table S.10 in the supplementary material.

## Discussion

The present Registered Report was designed to assess whether our new Cognitive and Affective Discounting (CAD) paradigm is suitable for determining individual subjective values of the ER strategies distraction, distancing, and suppression. We adapted Westbrook et al.’s^29^ Cognitive Effort Discounting paradigm in a way that allows SVs to be determined for tasks without objective difficulty order. The new paradigm was tested on an *n*-back task^34^ and a classic ER paradigm. The latter was the purpose of the present study and was completed by $N=120$ participants. As expected, the use of ER strategies was associated with reduced subjective and physiological arousal. This finding is in line with previous meta-analytic findings indicating the effectiveness of ER strategies, both on subjective as well as physiological levels^4,5^. Furthermore, we found higher levels of subjective cognitive effort for all ER strategies compared to active viewing. This allows us to replicate previous findings from our research group, showing that strategy use is associated with cognitive effort^22^. Taken together, these findings show that the ER strategies had the intended effect on the participants: Individuals were able to effectively reduce subjective and physiological responding at the expense of cognitive effort. Despite these distinct effects in the manipulation checks, the arousal and effort measures showed high variability between individuals, emphasizing the great extent of subjectivity when dealing with emotional stimuli. Additionally, it was surprising that the strategy suppression showed the lowest *corrugator* activity, the lowest effort ratings, and the highest utility ratings. In the case of the EMG measurement, this could be due to the fact that the result of the implementation of the instruction (“Maintain a neutral facial expression”) is measured directly, which also reduces the complexity of the generation process. This considerable degree of immediacy and simplicity might not only reduce the subjective effort, but might also increase the subjective utility of the strategy suppression. In addition, the participants receive relatively direct feedback from their own facial muscle activity as to how well suppression has been implemented, which likely influences their perceived regulation success. In contrast, the strategies distraction and distancing require a more detailed evaluation of internal states in order to assess their utility and success, which in turn requires more effort.

Almost all participants showed an absolute preference for a particular strategy over the two others, indicated by an SV of 1. We also found a wide range of SVs (between 0.005 and 1.00) across the whole sample, suggesting that individuals have varying degrees of preference strength. But despite this variation, most participants chose the strategy to which they had assigned their highest SV, supporting hypothesis H7a. We also found associations between individual SVs and various predictors. Subjective effort, utility, and *corrugator* muscle activity significantly predicted individual SVs across all strategies. Contrary to our hypothesis H6, utility but not effort was the best predictor for individual SVs, explaining 15.5% of variance in SVs. However, since individual SVs did not show associations with self-reported ER flexibility, we found no evidence for hypothesis H7b. In a subsequent exploratory analyses, we found a positive association between individual SVs and self-control. This is consistent with the literature, which has already reported correlations between self-control and demand avoidance^69^. However, we did not find an association between NFC and SVs. This is in contrast to reported correlations between NFC, effort discounting, and demand avoidance in cognitive tasks^29,34^. The role of NFC in affective tasks is not well understood yet.

### Ecological validity of subjective values of ER strategies

Our aim was to calculate individual subjective values in order to develop a better understanding of ER strategy selection. Most individuals show large variability in strategy choice, both within-strategy and between-strategy^17,70,71^, which in the context of emotion regulation is most likely a sign of good adaptability^12,17^. In addition, a variety of factors that influence strategy choice in specific situations have been examined^20–22,72–74^, including situation intensity and effort. However, these factors have often been studied in isolation from each other, and only rarely in conjunction^73^. Furthermore, the usual paradigms used in ER choice research (e.g., Sheppes et al.^21^) can only estimate how a factor tends to drive the choice in one direction or the other. They cannot determine the internal subjective value individuals attribute to all choice options. We are confident that we have achieved this with the present paradigm. We were not only able to show which factors have an influence on the values, but we were also able to demonstrate the values’ practical relevance in the form of choice prediction. As an operationalization of ER effectiveness, *corrugator* activity showed a significant association with SVs, but neither *levator* activity or subjective arousal did. With regard to the EMG measures, this could be because all the pictures we used were negative, which is commonly associated with higher *corrugator* activity, but only a small proportion of the pictures were classified as disgusting and thus elicited relatively specific *levator* activity. However, *corrugator* activity did not differ significantly between ER strategies, but was still associated with SVs. One possible reason for this could be that muscle activity provides direct feedback on the effectiveness of the current strategy in a more immediate fashion than, for example, the subjective arousal rating at the end of each experimental block. Furthermore, the finding that effort was associated with SVs confirms previous research by our group showing that individuals strive to minimise effort when choosing ER strategies^22^. Finally, the subjective utility ratings showed the greatest explained variance in the SVs. This relationship is highly plausible as it involves individuals assessing the utility of the strategy as a means of achieving external and internal regulatory goals. Utility is likely to overlap with subjective values - some literature even argues that utility and subjective values are one and the same^75^. However, this claim is not supported by our data, as subjective utility could only explain 15.5% of the variance in SVs, which leaves a considerable portion of variance in SVs unaccounted for.

The highest SV of each participant was associated with the choice made in the last experimental block. So far, it has been difficult to transfer such findings from the laboratory to everyday life^72^, likely because laboratory studies provide predefined and limited choice options in their experimental design^20–22^, which is not the case in a natural setting. Therefore, previous studies have attempted to investigate ER choice and its influencing factors in everyday life. But despite covering a large part of the emotion generation process^2^, even these studies prescribed certain strategies (for example studies see English et al.^76^, Millgram et al.^77^, Wilms et al.^72^). Similarly, the calculation of SVs in our new CAD paradigm depends on the available choice options that were defined in the experimental design. To allow all strategies in the ER repertoire to be recorded for each individual, a study might use ecological momentary assessment^12,78^. This would also capture strategies that are rarely used or are even considered maladaptive, such as alcohol consumption or rumination^79^.

In order to gain a more comprehensive picture of ER, dynamic or cyclic processes have to be considered. The extended process model of emotion regulation^33^ postulates three sequential stages, namely identification, selection, and implementation, to achieve a given goal in a situation. If the regulatory goal is not achieved, the ER strategy can be maintained, switched, or stopped^33^. Importantly, feedback on the success of implementing an ER strategy influences the choice of ER strategies in future situations, because the regulation context is changed through contextual feedback^33,80–82^. This means that studies on ER Choice should consider not only situational factors (i.e., perceived control, emotional intensity^72^), but also contextual factors (i.e., state-dependent psychological processes of the participant and task characteristics; for a review, see Aldao, 2013^83^)^81^. In a classic ER choice paradigm^21^, Murphy and Young^81^ could show that strategy choice was significantly influenced by both strategy choice and negative affect during the previous trial, providing evidence that experience gained during the use of ER strategies influences the future choice of ER strategies. Our newly developed CAD paradigm also makes an important contribution here. The structure of the paradigm provides the opportunity for participants’ experiences to influence their SVs, because each participant completes all ER strategies before indicating their preferences the discounting procedure, expecting to be re-applying one of the strategies at the end.

### Trait character of SVs

Knowing whether the SVs of ER strategies have a trait character would allow a further evaluation of their practical relevance and predictive power. With the data of the present study, a trait analysis is not possible, because the SVs of the ER strategies were assessed in only one situation at only one time point, which by definition represents a state. A habit would imply consistency of SVs across time points in similar situations, whereas a trait would imply consistency across both time points and situations. As noted above, ER choice behaviour is rather state-like, because it is influenced by personal regulatory goals, situational factors, and contextual demands^21^. We therefore believe that the influence of these factors on ER behaviour will also translate into state- or habit-like properties of SVs. Such factors could be varied systematically in order to shed light on the stability of SVs, e.g. by manipulating situational factors such as stimulus intensity, or by systematically assessing the goals that participants pursue with their ER behaviour. As Wilms and colleagues^72^ pointed out, situational factors and ER goals are state-like themselves, because they vary greatly across time points and situations. While participants in the lab mainly pursue hedonic but not social goals^85^, a real-life situation with social goals is likely to change not only their ER behaviour but also the SVs they assign to different strategies, especially when their choice options are not restricted by the experimental design (see also Limitations). To investigate whether the SVs of ER strategies can be conceptualised as states, habits, or traits, one could employ latent state trait modelling, as recently done by our group in a related context^30^. A systematic (non-)variation of situational factors and the assessment of personal factors, e.g. ER goals, can then help to disentangle time- and situation-specific variance in SVs. Importantly, the practical relevance and predictive power of SVs should be assessed at every measurement, as it is quite possible that the correlation between SVs and ER choice is situation-specific as well. Such findings would not only provide important insights into ER behaviour, but allow investigations into the association of ER behaviour with external criteria as well, such as well-being^9^.

### Limitations

A number of limitations must be taken into account when considering our findings. First, it should be noted that a block design was used, which might have resulted in habituation effects of EMG activity within the block. However, block designs are common in ER research^86^ and have been used in previous studies^87^. Secondly, it should be mentioned that subjective arousal, effort, and utility ratings were made retrospectively at the end of each block, which might have led to recency effects. But since it is known that affect labeling can attenuate emotional experience^88,89^, we decided not to conduct ratings after each image. Furthermore, we were able to confirm that the implementation of ER strategies was successful on both subjective and physiological levels. Still, these features of our research design may have led to slightly lower associations between SVs and predictors.

Third, a major limitation is that participants had to use three prescribed ER strategies. It may be that some of the participants were not used to any of these strategies in everyday life, so none of the strategies actually had a high subjective value for them. However, the strategies selected for attentional deployment, cognitive change, and response modulation have been shown meta-analytically to be most effective^4^. In this context, the individual SVs of each person must be interpreted with caution. They depend on the specific context: The stimuli presented and the strategies compared. For example, SVs for an ER strategy might be higher or lower when different stimuli or stimulus valences and different comparison strategies are used, because the calculation of SVs is inseparable from the other SVs.

Fourth, the highest value during the discounting paradigm was set to 2 € as fixed value. Participants were asked to imagine that this was the amount of money they would receive if they repeated this strategy. Thus, 2 € could be an insufficient incentive to repeat a whole experimental block. However, we chose this amount because we wanted to follow the original paradigm of Westbrook^29^, and because it has been shown that a lower incentive increases participants’ sensitivity to effort differences^90^. In the future, however, it should be investigated how the incentive size affects subjective values.

### Conclusion

In order to cope with changing emotional demands, individuals may flexibly select and apply ER strategies from their repertoire^12,13^. They select the strategy that is most suitable for coping with contextual demands and achieving regulatory goals^12,85^. The combination of influencing factors should be reflected in subjective values that are formed for all alternatives and serve as a basis for decision-making. To date, such subjective values have not been established for ER strategies. Our proposed CAD paradigm contributes to research on ER Choice and ER Flexibility by allowing quantification of these values. This further enables to investigate the factors influencing the internal generation of these subjective values of ER strategies in more detail. It appears that the subjective value attributed to a strategy is primarily determined by perceived usefulness and effort. Finally, further research is needed to investigate the factors that influence subjective values and whether these values represent habitual use of ER strategies by individuals.

### Supplementary Information

Supplementary Information.

## Data Availability

The data of this study can be downloaded from osf.io/vnj8x/.

## Code availability

The paradigm code, the R script for analysis, and the R Markdown file used to compile this document are available at osf.io/vnj8x/.
