# Supplementary material for: Estimating individual subjective values of emotion regulation strategies
Source: Sci Rep. 2023 Aug 15;13:13262. doi: 10.1038/s41598-023-40034-7 (PMC10427653; doi:10.1038/s41598-023-40034-7)
Supplement: Supplementary file 1 — Supplementary Information. [file 41598_2023_40034_MOESM1_ESM.pdf]

Supplementary Material for the Registered Report: Estimating individual subjective values of emotion regulation strategies

Christoph Scheffel[†,1], Josephine Zerna[†,1], Anne Gärtner[1], Denise Dörfel[1,2], & Alexander Strobel[1]

[1] Chair of Differential and Personality Psychology, Faculty of Psychology, Technische Universität Dresden, 01069 Dresden, Germany

[2] Center for Information Services and High Performance Computing, Technische Universität Dresden, 01069 Dresden, Germany

Author Note

The authors made the following contributions. Christoph Scheffel: Conceptualization, Methodology, Funding acquisition, Formal analysis, Investigation, Project administration, Software, Visualization, Writing - original draft preparation, Writing - review & editing; Josephine Zerna: Conceptualization, Methodology, Funding acquisition, Investigation, Project administration, Software, Visualization, Writing - review & editing; Anne Gärtner: Formal analysis, Writing - review & editing; Denise Dörfel: Conceptualization, Writing - review & editing; Alexander Strobel: Conceptualization, Methodology, Writing - review & editing. † Christoph Scheffel and Josephine Zerna contributed equally to this work.

Correspondence concerning this article should be addressed to Christoph Scheffel, Zellescher Weg 17, 01069 Dresden, Germany. E-mail: christoph_scheffel@tu-dresden.de

Supplementary Material for the Registered Report: Estimating individual subjective values of emotion regulation strategies

Design Table

**Question**	**Hypothesis**	**Sampling plan (e.g. power analysis)**	**Analysis Plan**	**Interpretation given to different outcomes**
1.) Do negative pictures (compared to neutral pictures) evoke subjective arousal and physiological responding? (Manipulation check)	1a) Subjective arousal (arousal rating) is lower after actively viewing neutral pictures compared to actively viewing negative pictures.	F tests - ANOVA: Repeated measures, within factors<br>Analysis: A priori: Compute required sample size<br>Input:<br>Effect size f = 1.59 (η_p^2 = 0.716) (Scheffel et al., 2021)<br>α err prob = 0.05<br>Power (1-β err prob) = 0.95<br>Number of groups = 1<br>Number of measurements = 2<br>Corr among rep measures = 0.5<br>Nonsphericity correction ε = 1<br><br>Output:<br>Noncentrality parameter λ = 40.3380260<br>Critical F = 10.1279645<br>Numerator df = 1.0<br>Denominator df = 3.0<br>Total sample size = 4<br>Actual power = 0.9789865	Repeated measures ANOVA with two linear contrasts, comparing the subjective arousal ratings of two blocks (active viewing – neutral and active viewing - negative).<br><br>ANOVA is calculated using aov_ez() function of the afex-package, estimated marginal means are calculated using emmeans() function from the emmeans-package: if the factor Block is significant, pairwise contrasts are calculated using pairs() with Bonferroni adjustment for multiple testing.<br><br>Bayes factors are computed for the ANOVA and each contrast using the BayesFactor-package.	ANOVA yields $p < .05$ is interpreted as subjective arousal (arousal ratings) changing significantly with blocks. Values of arousal ratings are interpreted as equal between blocks if $p > .05$.<br><br>Each contrast yielding $p < .05$ is interpreted as arousal ratings being different between those two blocks, magnitude and direction are inferred from the respective estimate. Values of arousal ratings are interpreted as equal between blocks if $p > .05$.<br><br>The Bayes factor *BF10* is reported alongside every *p*-value to assess the strength of evidence.
	1b) Physiological responding (EMG *corrugator* activity) is lower while actively viewing neutral pictures compared to actively viewing negative pictures.	F tests - ANOVA: Repeated measures, within factors<br>Analysis: A priori: Compute required sample size<br>Input:<br>Effect size f = 0.5573293 (η_p^2 = 0.237) (Pilot Study)<br>α err prob = 0.05<br>Power (1-β err prob) = 0.95<br>Number of groups = 1<br>Number of measurements = 2	Repeated measures ANOVA with two linear contrasts, comparing the EMG *corrugator* activity of two blocks (active viewing – neutral and active viewing - negative).<br><br>ANOVA is calculated using aov_ez() function of the afex-package, estimated marginal means are calculated using	ANOVA yields $p < .05$ is interpreted as physiological responding (EMG *corrugator* activity) changing significantly with blocks. Values of EMG *corrugator* activity are interpreted as equal between blocks if $p > .05$.<br><br>Each contrast yielding $p < .05$ is interpreted as EMG *corrugator* activity being different between those two blocks, magnitude and direction are

		Corr among rep measures = 0.5<br>Nonsphericity correction ε = 1<br><br><u>Output</u>:<br>Noncentrality parameter λ = 16.1520293<br>Critical F = 4.7472253<br>Numerator df = 1.0<br>Denominator df = 12.0<br>Total sample size = 13<br>Actual power = 0.9573615	emmeans() function from the emmeans-package: if the factor Block is significant, pairwise contrasts are calculated using pairs() with Bonferroni adjustment for multiple testing.<br><br>Bayes factors are computed for the ANOVA and each contrast using the BayesFactor-package.	inferred from the respective estimate. Values of EMG *corrugator* activity are interpreted as equal between blocks if $p > .05$.<br><br>The Bayes factor *BF10* is reported alongside every *p*-value to assess the strength of evidence.
	1c) Physiological responding (EMG *levator* activity) is lower while actively viewing neutral pictures compared to actively viewing negative pictures.	F tests - ANOVA: Repeated measures, within factors<br>Analysis: A priori: Compute required sample size<br><u>Input:</u><br>Effect size f = 0.4396788 (η_p^2 = 0.162) (Pilot Study)<br>α err prob = 0.05<br>Power (1-β err prob) = 0.95<br>Number of groups = 1<br>Number of measurements = 2<br>Corr among rep measures = 0.5<br>Nonsphericity correction ε = 1<br><br><u>Output</u>:<br>Noncentrality parameter λ = 14.6921260<br>Critical F = 4.4138734<br>Numerator df = 1.0<br>Denominator df = 18.0<br>Total sample size = 19<br>Actual power = 0.9517060	Repeated measures ANOVA with two linear contrasts, comparing the EMG *levator* activity of two blocks (active viewing – neutral and active viewing - negative).<br><br>ANOVA is calculated using aov_ez() function of the afex-package, estimated marginal means are calculated using emmeans() function from the emmeans-package: if the factor Block is significant, pairwise contrasts are calculated using pairs() with Bonferroni adjustment for multiple testing.<br><br>Bayes factors are computed for the ANOVA and each contrast using the BayesFactor-package.	ANOVA yields $p < .05$ is interpreted as physiological responding (EMG *levator* activity) changing significantly with blocks. Values of EMG *levator* activity are interpreted as equal between blocks if $p > .05$.<br><br>Each contrast yielding $p < .05$ is interpreted as EMG *levator* activity being different between those two blocks, magnitude and direction are inferred from the respective estimate. Values of EMG *levator* activity are interpreted as equal between blocks if $p > .05$.<br><br>The Bayes factor *BF10* is reported alongside every *p*-value to assess the strength of evidence.

2.) Do ER strategies reduce emotional arousal? (Manipulation check)	2a) Subjective arousal (arousal rating) is lower after using an emotion regulation strategy (distraction, distancing, suppression) compared to active viewing.	F tests - ANOVA: Repeated measures, within factors<br>Analysis: A priori: Compute required sample size<br>Input:<br>Effect size f = 0.50 (η_p^2 = 0.20) (Scheffel et al., 2021)<br>α err prob = 0.05<br>Power (1-β err prob) = 0.95<br>Number of groups = 1<br>Number of measurements = 4<br>Corr among rep measures = 0.5<br>Nonsphericity correction ε = 1<br><br>Output:<br>Noncentrality parameter λ = 20.0<br>Critical F = 2.9603513<br>Numerator df = 3.0<br>Denominator df = 27.0<br>Total sample size = 10<br>Actual power = 0.95210128	Repeated measures ANOVA comparing the subjective arousal ratings of four blocks (active viewing, distraction, distancing, suppression).<br><br>ANOVA is calculated using aov_ez() function of the afex-package, estimated marginal means are calculated using emmeans() function from the emmeans-package: if the factor Block is significant, pairwise contrasts are calculated using pairs() with Bonferroni adjustment for multiple testing.<br><br>Bayes factors are computed for the ANOVA and each contrast using the BayesFactor-package.	ANOVA yields *p* < .05 is interpreted as arousal ratings changing significantly with blocks. Values of arousal ratings are interpreted as equal between blocks if *p* > .05.<br><br>Each contrast yielding *p* < .05 is interpreted as arousal ratings being different between those two blocks, magnitude and direction are inferred from the respective estimate. Values of arousal ratings are interpreted as equal between blocks if *p* > .05.<br><br>The Bayes factor *BF10* is reported alongside every *p*-value to assess the strength of evidence.
3.) Do ER strategies reduce physiological responding? (Manipulation check)	3a) Physiological responding (EMG *corrugator* activity) is lower after using an emotion regulation strategy (distraction, distancing, suppression) compared to active viewing.	F tests - ANOVA: Repeated measures, within factors<br>Analysis: A priori: Compute required sample size<br>Input:<br>Effect size f = 0.1605 (Zaehringer et al., 2020)<br>α err prob = 0.05<br>Power (1-β err prob) = 0.95<br>Number of groups = 1<br>Number of measurements = 4<br>Corr among rep measures = 0.5<br>Nonsphericity correction ε = 1	Repeated measures ANOVA comparing the *corrugator* muscle activity of four blocks (active viewing, distraction, distancing, suppression).<br><br>ANOVA is calculated using aov_ez() function of the afex-package, estimated marginal means are calculated using emmeans() function from the emmeans-package: if the factor Block is significant, pairwise contrasts are calculated using	ANOVA yields *p* < .05 is interpreted as *corrugator* muscle activity changing significantly with blocks. Values of *corrugator* muscle activity are interpreted as equal between blocks if *p* > .05.<br><br>Each contrast yielding *p* < .05 is interpreted as *corrugator* muscle activity being different between those two blocks, magnitude and direction are inferred from the respective estimate. Values of *corrugator* muscle activity

		<u>Output</u>:<br>Noncentrality parameter λ = 17.5169700<br>Critical F = 2.6404222<br>Numerator df = 3.0<br>Denominator df = 252<br>Total sample size = 85<br>Actual power = 0.9509128	pairs() with Bonferroni adjustment for multiple testing.<br><br>Bayes factors are computed for the ANOVA and each contrast using the BayesFactor-package.	are interpreted as equal between blocks if $p > .05$.<br><br>The Bayes factor *BF10* is reported alongside every *p*-value to assess the strength of evidence.
	3b) Physiological responding (EMG *levator* activity) is lower after using an emotion regulation strategy (distraction, distancing, suppression) compared to active viewing.	F tests - ANOVA: Repeated measures, within factors<br>Analysis: A priori: Compute required sample size<br><u>Input:</u><br>Effect size f = 0.1605 (Zaehringer et al., 2020)<br>α err prob = 0.05<br>Power (1-β err prob) = 0.95<br>Number of groups = 1<br>Number of measurements = 4<br>Corr among rep measures = 0.5<br>Nonsphericity correction ε = 1<br><br><u>Output</u>:<br>Noncentrality parameter λ = 17.5169700<br>Critical F = 2.6404222<br>Numerator df = 3.0<br>Denominator df = 252<br>Total sample size = 85<br>Actual power = 0.9509128	Repeated measures ANOVA comparing the *levator* muscle activity of four blocks (active viewing, distraction, distancing, suppression).<br><br>ANOVA is calculated using aov_ez() function of the afex-package, estimated marginal means are calculated using emmeans() function from the emmeans-package: if the factor Block is significant, pairwise contrasts are calculated using pairs() with Bonferroni adjustment for multiple testing.<br><br>Bayes factors are computed for the ANOVA and each contrast using the BayesFactor-package.	ANOVA yields $p < .05$ is interpreted as *levator* muscle activity changing significantly with blocks. Values of *levator* muscle activity are interpreted as equal between blocks if $p > .05$.<br><br>Each contrast yielding $p < .05$ is interpreted as *levator* muscle activity being different between those two blocks, magnitude and direction are inferred from the respective estimate. Values of *levator* muscle activity are interpreted as equal between blocks if $p > .05$.<br><br>The Bayes factor *BF10* is reported alongside every *p*-value to assess the strength of evidence.
4.) Do ER strategies require cognitive effort? (Manipulation check)	4a) Subjective effort (effort rating) is greater after using an emotion regulation strategy (distraction,	F tests - ANOVA: Repeated measures, within factors<br>Analysis: A priori: Compute required sample size<br><u>Input:</u>	Repeated measures ANOVA comparing the subjective effort ratings of four blocks (active viewing, distraction, distancing, suppression).	ANOVA yields $p < .05$ is interpreted as effort ratings changing significantly with blocks. Values of effort ratings are interpreted as equal between blocks if $p > .05$.

	distancing, suppression) compared to active viewing.	Effect size f = 0.2041241 (η_p^2 = 0.04) (Scheffel et al., 2021)<br>α err prob = 0.05<br>Power (1-β err prob) = 0.95<br>Number of groups = 1<br>Number of measurements = 4<br>Corr among rep measures = 0.5<br>Nonsphericity correction ε = 1<br><br>Output:<br>Noncentrality parameter λ = 17.6666588<br>Critical F = 2.6625685<br>Numerator df = 3.0<br>Denominator df = 156.0<br>Total sample size = 53<br>Actual power = 0.95206921	ANOVA is calculated using aov_ez() function of the afex-package, estimated marginal means are calculated using emmeans() function from the emmeans-package: if the factor Block is significant, pairwise contrasts are calculated using pairs() with Bonferroni adjustment for multiple testing.<br><br>Bayes factors are computed for the ANOVA and each contrast using the BayesFactor-package.	Each contrast yielding $p < .05$ is interpreted as effort ratings being different between those two blocks, magnitude and direction are inferred from the respective estimate. Values of effort ratings are interpreted as equal between blocks if $p > .05$.<br><br>The Bayes factor *BF10* is reported alongside every *p*-value to assess the strength of evidence.
	4b) Majority of participants reuse the strategy that was least effortful for them.	-	Subjects are asked about the reasons for their choice in the follow-up survey. These answers are classified into categories and counted.	The percentage choice of strategies is described descriptively.
5.) Which variables can predict individual subjective values of ER strategies?	5a) Subjective effort (effort ratings) negatively predict subjective values of ER strategies.	t tests - Linear multiple regression: Fixed model, single regression coefficient<br>Analysis: A priori: Compute required sample size<br>Input:<br>Tail(s) = One<br>Effect size f^2 = 0.34 (Since there are no findings in this respect yet,	Multilevel model of SVs with level-1-predictors subjective effort, subjective arousal, subjective utility, *corrugator*, and *levator* muscle activity using subject specific intercepts and allowing random slopes for ER strategies.	Fixed effects yield $p < .05$ are interpreted as subjective values are related to subjective effort. Subjective values are interpreted as not being related to subjective effort if $p > .05$.<br><br>The Bayes factor *BF10* is reported alongside every *p*-value to assess the strength of evidence.

	5b) Subjective arousal (arousal ratings) negatively predict subjective values of ER strategies.	we have inferred from the effect size in the closest-similar model: Westbrook et al., 2013)<br>α err prob = 0.05<br>Power (1-β err prob) = 0.95<br>Number of predictors = 4<br>Output:<br>Noncentrality parameter δ = 3.4<br>Critical t = 1.6991270<br>Df = 29<br>Total sample size = 34<br>Actual power = 0.9529571	The null model and the random slopes model are calculated using lmer() of the lmerTest-package.<br><br>Bayes factors are computed for the MLM using the BayesFactor-package.	Fixed effects yield $p < .05$ are interpreted as subjective values are related to subjective arousal. Subjective values are interpreted as not being related to subjective arousal if $p > .05$.<br><br>The Bayes factor *BF10* is reported alongside every *p*-value to assess the strength of evidence.
	5c) Subjective utility (utility ratings) positively predict subjective values of ER strategies.			Fixed effects yield $p < .05$ are interpreted as subjective values are related to subjective utility. Subjective values are interpreted as not being related to subjective utility if $p > .05$.<br><br>The Bayes factor *BF10* is reported alongside every *p*-value to assess the strength of evidence.
	5d) Physiological responding (EMG *corrugator* activity) negatively predict subjective values of ER strategies.			Fixed effects yield $p < .05$ are interpreted as subjective values are related to *corrugator* activity. Subjective values are interpreted as not being related to *corrugator* activity if $p > .05$.<br><br>The Bayes factor *BF10* is reported alongside every *p*-value to assess the strength of evidence.
	5e) Physiological responding (EMG *levator* activity) negatively predict subjective values of ER strategies.			Fixed effects yield $p < .05$ are interpreted as subjective values are related to *levator* activity. Subjective values are interpreted as not being related to *levator* activity if $p > .05$.

				The Bayes factor *BF10* is reported alongside every *p*-value to assess the strength of evidence.
6.) Is the effort required for an ER strategy the best predictor for subjective values of ER strategies?	6a) Subjective values decline with increasing effort, even after controlling for task performance (subjective arousal ratings), utility (subjective utility ratings), and physiological responding (EMG *corrugator* and *levator* activity).	t tests - Linear multiple regression: Fixed model, single regression coefficient<br>Analysis: A priori: Compute required sample size<br>Input:<br>Tail(s) = One<br>Effect size $f^2 = 0.34$ (Since there are no findings in this respect yet, we have inferred from the effect size in the closest-similar model: Westbrook et al., 2013)<br>α err prob = 0.05<br>Power (1-β err prob) = 0.95<br>Number of predictors = 4<br>Output:<br>Noncentrality parameter $\delta = 3.4$<br>Critical t = 1.6991270<br>Df = 29<br>Total sample size = 34<br>Actual power = 0.9529571		Fixed effects yield $p < .05$ are interpreted as subjective values changing significantly with ER strategy. Subjective values are interpreted as equal between ER strategies if $p > .05$.<br>The Bayes factor *BF10* is reported alongside every *p*-value to assess the strength of evidence.
7.) Are subjective values related to flexible emotion regulation?	7a) The higher the subjective value, the more likely the respective strategy is chosen.	1) χ^2 tests – Goodness-of-fit tests_ Contingency tables<br>Analysis: A priori: Compute required sample size<br>Input:<br>Effect size $\omega = 0.5$ (Based on our theoretical considerations, we assume a large effect)<br>α err prob = 0.05	1) Chi-squared test with the variables “predicted choice” (= highest SV of each participant) and “choice” (Strategy 1, 2, or 3)<br>2) Ordinal regression with dependent variable “Choice” (Strategy 1, 2, or 3) and independent variables “SV	1) χ^2 yields $p < .05$ is interpreted as predicted choice (highest SV of each participant) and actual choice show significant consistency.<br>Predicted choice and actual choice are interpreted as independent if $p > .05$.

		Power (1-β err prob) = 0.95<br>Df = 1<br><u>Output:</u><br>Noncentrality parameter λ = 19.8<br>Critical χ^2 = 11.0704977<br>Total sample size = 52<br>Actual power = 0.9500756<br><br>2) z tests –Logistic regression<br>Analysis: A priori: Compute required sample size<br><u>Input:</u><br>Tails: One<br>Pr(Y=1|X=1) H1 = 0.80 (Based on our theoretical considerations, that a higher SVs should lead almost certainly to the choice of the respective strategy)<br>Pr(Y=1|X=1) H0 = 0.333 (Based on theoretical considerations: if all SVs are equal, choice is on chance level)<br>α err prob = 0.05<br>Power (1-β err prob) = 0.95<br>R^2 other X = 0<br>X distribution: normal<br>X param μ = 0<br>X param σ = 1<br><u>Output:</u><br>Critical z = 1.6448536<br>Total sample size = 25<br>Actual power = 0.9528726	strategy 1", "SV strategy 2" and "SV strategy 3".	The Bayes factor *BF10* is reported alongside every *p*-value to assess the strength of evidence.<br><br>2) Ordinal logistic regression yields $p <$ .05 is interpreted as the respective subjective value has a significant influence on the OR of the choice of a strategy.<br>Respective SV is interpreted as not related to choice if $p > .05$.<br><br>The Bayes factor *BF10* is reported alongside every *p*-value to assess the strength of evidence.

	7b) Subjective values are lower and decline stronger when ER flexibility is lower.	t tests – Linear multiple regression: Fixed model, single regression coefficient<br>Analysis: A priori: compute required sample size<br><u>Input:</u><br>Tail(s) = One<br>Effect size $f^2 = 0.15$ (as there is no evidence in the literature, we assume a medium sized effect)<br>α err prob = 0.05<br>Power (1-β err prob) = 0.95<br>Number of predictors = 2<br><u>Output:</u><br>Noncentrality parameter δ = 3.316662<br>Critical t = 1.69665997<br>Df = 71<br>Total sample size = 74<br>Actual power = 0.95101851	SVs will be sorted by magnitude in descending order. Values will be fitted in a linear model to estimate the individual intercept (i.e., the extent to which an individual considers any of the ER strategies useful) and slope (i.e., the extent to which one strategy is preferred over others, indicating less flexibility).<br><br>A linear regression will be computed with individual intercepts and slopes as predictors and FlexER score as criterion.	β yield $p < .05$ are interpreted as significant association between predictor (intercept, slope) and ER flexibility. The direction of effect is interpreted according to sign (negative or positive). *p* – values > .05 are interpreted as no association between predictor and ER flexibility.<br><br>The Bayes factor *BF10* is reported alongside every *p*-value to assess the strength of evidence.

Exploratory: Are individual subjective values of ER strategies related to personality traits?			Multilevel model of SVs with level-1-predictors subjective effort, subjective arousal, *corrugator*, and *levator* muscle activity and level-2-predictors NFC and self-control using subject specific intercepts and allowing random slopes for ER strategies.<br><br>The null model and the random slopes model are calculated using lmer() of the lmerTest-package.<br><br>Bayes factors are computed for the MLM using the BayesFactor-package.	Fixed effects yield $p < .05$ are interpreted as subjective values are related to NFC and self-control. Subjective values are interpreted as not being related to subjective effort if $p >$ .05.<br><br>The Bayes factor *BF10* is reported alongside every *p*-value to assess the strength of evidence.

Stimuli used in ER paradigm

Table S1

List of IAPS (Lang, Bradley, and Cuthbert, 2008) and EmoPicS (Wessa et al., 2010) used in the ER paradigm.

	Neutral	Negative 1	Negative 2	Negative 3	Negative 4	Negative 5
	083†	225†	210†	208†	227†	223†
	107†	230†	218†	219†	252†	238†
	124†	255†	222†	226†	1051*	245†
	140†	327†	228†	253†	2800*	2981*
	143†	1111*	246†	254†	3061*	3016*
	7000*	3017*	251†	326†	3230*	3101*
	7002*	3022*	2703*	1301*	6561*	3181*
	7004*	3180*	3051*	3350*	6838*	3215*
	7006*	3280*	3160*	6242*	9120*	3220*
	7009*	6190*	3185*	6410*	9181*	3225*
	7021*	6244*	3301*	6555*	9185*	6020*
	7025*	6836*	6562*	6825*	9230*	6571*
	7041*	9180*	9031*	6940*	9254*	6831*
	7100*	9182*	9040*	8230*	9295*	8231*
	7150*	9253*	9042*	9041*	9332*	9373*
	7185*	9300*	9043*	9140*	9411*	9400*
	7211*	9326*	9145*	9340*	9420*	9402*
	7224*	9424*	9160*	9409*	9421*	9403*
	7233*	9425*	9184*	9570*	9599*	9405*
	7235*	9920*	9904*	9800*	9905*	9423*
**Valence**	4.86 ± 0.49	2.84 ± 0.57	2.64 ± 0.46	2.82 ± 0.62	2.65 ± 0.75	2.74 ± 0.70
**Arousal**	3.01 ± 0.61	5.62 ± 0.34	5.58 ± 0.38	5.60 ± 0.39	5.61 ± 0.41	5.63 ± 0.37

Note. * Pictures taken from the IAPS (Lang, Bradley, and Cuthbert, 2008); † Pictures taken from the EmoPicS (Wessa et al., 2010).

Detailed information on psychometric measures

WHO-5. General psychological well-being was assessed using the WHO-5 scale[1,2]. Five items such as “Over the past 2 weeks I have felt calm and relaxed.” are rated on a 6-point Likert scale raning from 0 (at no time) to 5 (all of the time). The German version of the scale showed a high internal consistency (Cronbach’s $\alpha = .92$)[2].

Connor-Davidson Resilience Scale. Resilience was assessed using the Connor-Davidson Resilience Scale (CD-RISC)[3–5]. Ten items such as “I am able to adapt to change.” are rated on a scale from 0 (not true at all) to 4 (true nearly all the time). The 10-item version showed a high internal consistency (Cronbach’s $\alpha = .84$) and a satisfactory retest-reliability of $r_{tt} = .81$ across 6 months[5].

Emotion Regulation Questionnaire. Habitual use of reappraisal and suppression was measured using the 10-item Emotion Regulation Questionnaire (ERQ)[6,7]. The scale has items such as “I keep my emotions to myself” (ERQ-suppression - 4 items) and “When I’m faced with a stressful situation, I make myself think about it in a way that helps me stay calm” (ERQ-reappraisal - 6 items), which are answered on a 7-point Likert scale ranging from 1 (strongly disagree) to 7 (strongly agree), and has acceptable to high internal consistency (Cronbach’s $\alpha > .75$)[8].

FlexER Scale. Flexible use of ER strategies is assessed using the FlexER Scale[9] with items such as “If I want to feel less negative emotions, I have several strategies to achieve this.”, which are answered on a 4-point scale ranging from “strongly agree” to “strongly disagree”. Psychometric properties are currently under investigation.

Implicit Theories Questionnaire. Implicit theories of willpower in emotional control were assessed using the Implicit Theories Questionnaire of Bernecker and Job[10]. Four items such as “Having to control a strong emotion makes you exhausted and you are less able to manage your feelings right afterwards.” are rated on a 6-point scale ranging from 1 (fully agree) to 6 (do not agree at all). The questionnaire showed an internal consistency of

Cronbach's $\alpha = .87$[10].

Need for Cognition Scale. Need for Cognition (NFC) was assessed with the 16-item short version of the German NFC scale[11]. Responses to each item (e.g., "Thinking is not my idea of fun", recoded) are recorded on a 7-point Likert scale ranging from -3 (completely disagree) to +3 (completely agree) and are summed to the total NFC score. The scale shows comparably high internal consistency (Cronbach's $\alpha > .80$)[11,12] and a retest reliability of $r_{tt} = .83$ across 8 to 18 weeks[13].

Self-Regulation Scale. As one measure of self-control, the Self-Regulation Scale (SRS)[14] was used. The scale has 10 items (e.g., "It is difficult for me to suppress thoughts that interfere with what I need to do.", recoded) on a 4-point scale ranging from 1 (not at all true) to 4 (exactly true). It has high internal consistency (Cronbach's $\alpha > .80$)[14].

Brief Self-Control Scale. As a second measure of self-control, the Brief Self-Control Scale (BSCS)[15,16] was used. It comprises 13 items (e.g., "I am good at resisting temptations") with a 5-point rating scale ranging from 1 (not at all like me) to 5 (very much like me). The scale shows acceptable internal consistency (Cronbach's $\alpha = .81$)[16].

Barratt Impulsiveness Scale. As a third measure of self-control, the Barratt Impulsiveness Scale (BIS-11)[17,18] was used. Responses to each item (e.g., "I am self-controlled.", recoded) are assessed on a 4-point scale ranging from 1 (never/rarely) to 4 (almost always/always). An internal consistency of Cronbach's $\alpha = .74$ and a retest reliability of $r_{tt} = .56$ for General Impulsiveness and $r_{tt} = .66$ for Total Score across 6 month were reported[18].

Attentional Control Scale. Attentional control was measured using the Attentional Control Scale (ACS)[19] with items such as "My concentration is good even if there is music in the room around me". The 20 items are rated on a 4-point scale ranging from 1 (almost never) to 4 (always). An internal consistency of Cronbach's $\alpha = .88$ was reported[19].

Test for normal distribution of predictor variables

Table S.2

Results of Shapiro-Wilk test for normal distribution of subjective arousal and effort ratings for all strategies.

	M	*SD*	*W*	*p*
Arousal View Neu	26.629	39.116	0.677	<.001
Arousal View Neg	187.778	87.308	0.979	0.057
Arousal Distraction	158.129	92.492	0.972	0.014
Arousal Distancing	168.617	95.754	0.978	0.043
Arousal Suppression	163.957	87.165	0.980	0.073
Effort View Neu	18.147	27.372	0.651	<.001
Effort View Neg	49.396	62.262	0.740	<.001
Effort Distraction	208.465	96.149	0.983	0.132
Effort Distancing	158.259	99.505	0.969	0.007
Effort Suppression	189.800	92.338	0.983	0.123

Table S.3

Results of Shapiro-Wilk test for normal distribution of Corrugator and Levator activity for all strategies.

	M	*SD*	*W*	*p*
Corrugator View Neu	0.041	6.991	0.046	<.001
Corrugator View Neg	1.030	7.213	0.194	<.001
Corrugator Distraction	0.004	7.668	0.040	<.001
Corrugator Distancing	0.066	3.784	0.083	<.001
Corrugator Suppression	0.246	1.924	0.354	<.001
Levator View Neu	0.090	1.838	0.384	<.001
Levator View Neg	0.580	3.198	0.429	<.001
Levator Distraction	-0.050	1.157	0.520	<.001
Levator Distancing	-0.027	0.917	0.481	<.001
Levator Suppression	0.010	0.996	0.554	<.001

Post-hoc contrasts for effects of valence on subjective arousal and physiological responding

Table S.4
Post-hoc contrasts for effects of valence on subjective arousal ratings in the active viewing conditions.

Contrast	Estimate	*SE*	*df*	*t*	*p*	BF_{10}	η_p^2	95%*CI*
$View_{neutral} - View_{negative}$	-161.15	8.06	119.00	-20.00	<.001	3.22×10^{36}	0.77	[0.72, 1.00]

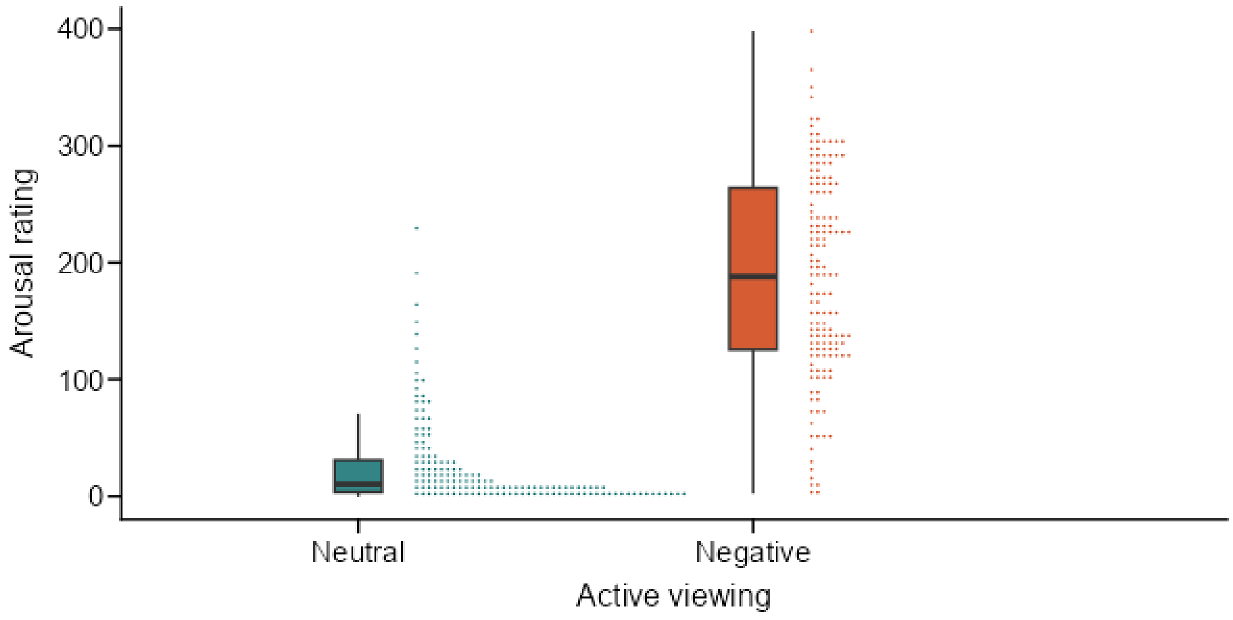

Figure S.1. Subjective arousal ratings of the active viewing conditions visualized as boxplots. Dots represent individual effort ratings placed in 150 quantiles.

Table S.5
Post-hoc contrasts for effects of valence on Corrugator activity in the active viewing conditions.

Contrast	Estimate	*SE*	*df*	*t*	*p*	BF_{10}	η_p^2	95%*CI*
$View_{neutral} - View_{negative}$	-0.27	0.05	117.00	-5.27	<.001	8.67×10^{16}	0.19	[0.10, 1.00]

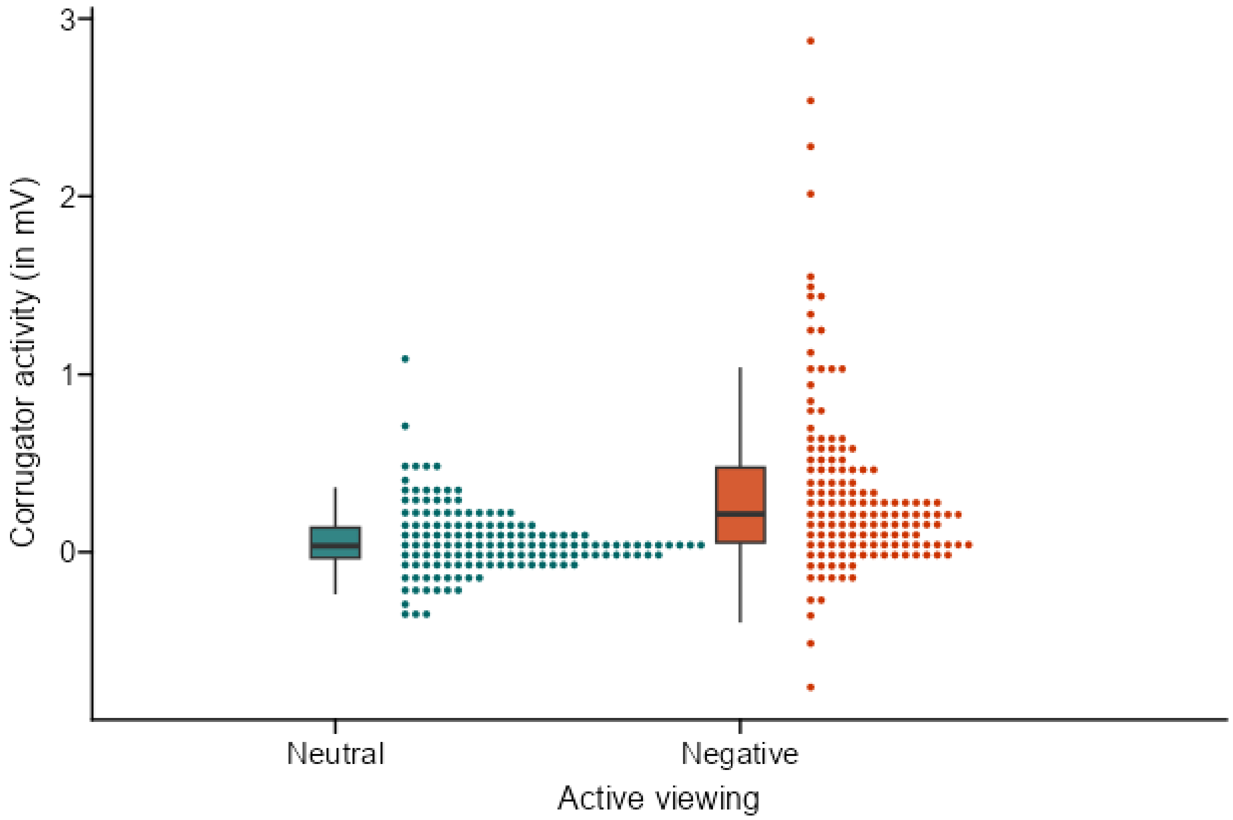

Figure S.2. Corrugator activity in mV during the active viewing conditions, visualized as boxplots. Dots represent individual Corrugator activity measures placed in 150 quantiles.

Table S.6

Post-hoc contrasts for effects of valence on Levator activity in the active viewing conditions.

Contrast	Estimate	*SE*	*df*	*t*	*p*	BF_{10}	η_p^2	95%*CI*
$View_{neutral} - View_{negative}$	-0.23	0.08	117.00	-2.98	<.001	188.72	0.07	[0.01, 1.00]

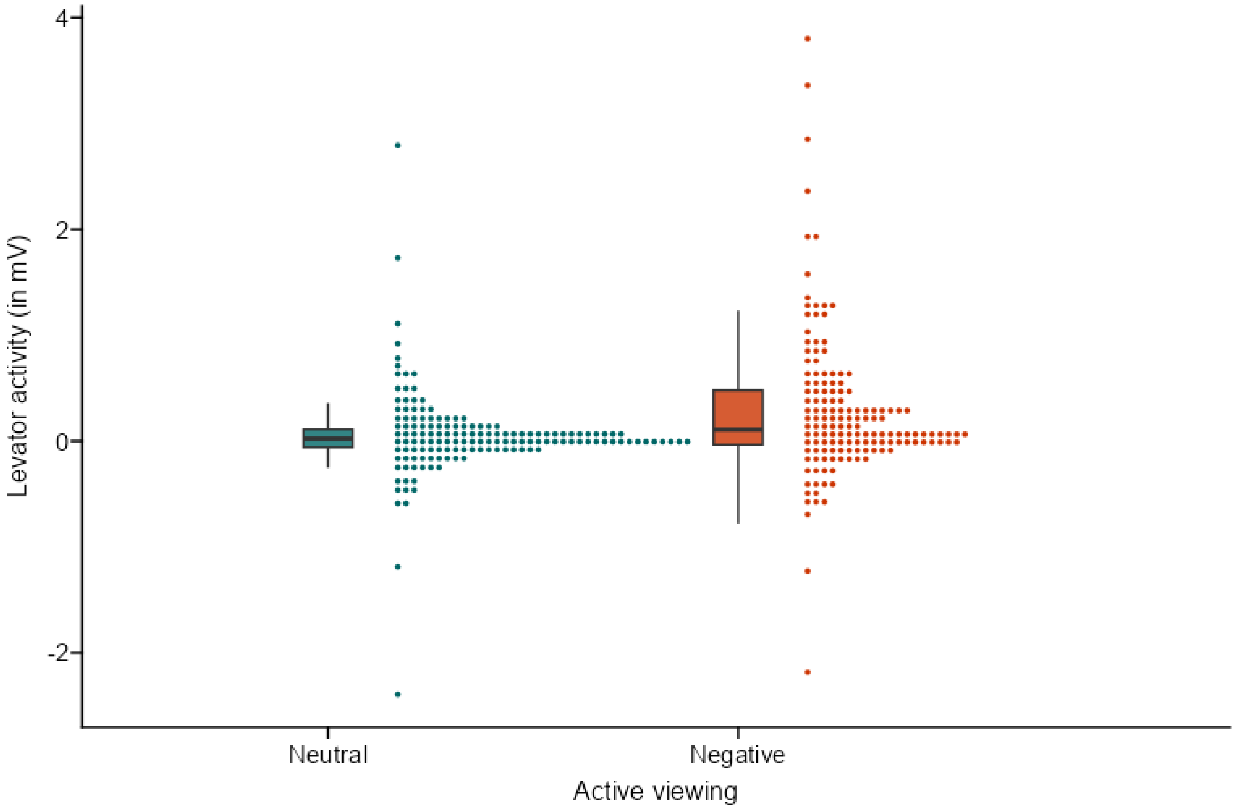

Figure S.3. Levator activity in mV during the active viewing conditions, visualized as boxplots. Dots represent individual Levator activity measures placed in 150 quantiles.

Post-hoc contrasts for effects of ER strategies on subjective arousal and physiological responding

Table S.7

Post-hoc contrasts for effects of ER strategies on subjective arousal ratings.

Contrast	Estimate	SE	df	t	p	$BF10$	η_p^2	95%CI
$View_{neg} - Distraction$	29.649	6.680	357.000	4.439	0.000	168.484	0.05	[0.02, 1.00]
$View_{neg} - Distancing$	23.820	6.680	357.000	3.566	0.002	62.990	0.03	[0.01, 1.00]
$View_{neg} - Suppression$	19.161	6.680	357.000	2.869	0.026	1.965	0.02	[0.00, 1.00]
$Distraction - Distancing$	-5.828	6.680	357.000	-0.873	1.000	0.179	2.13e-03	[0.00, 1.00]
$Distraction - Suppression$	-10.488	6.680	357.000	-1.570	0.704	0.309	6.86e-03	[0.00, 1.00]
$Distancing - Suppression$	-4.659	6.680	357.000	-0.698	1.000	0.135	1.36e-03	[0.00, 1.00]

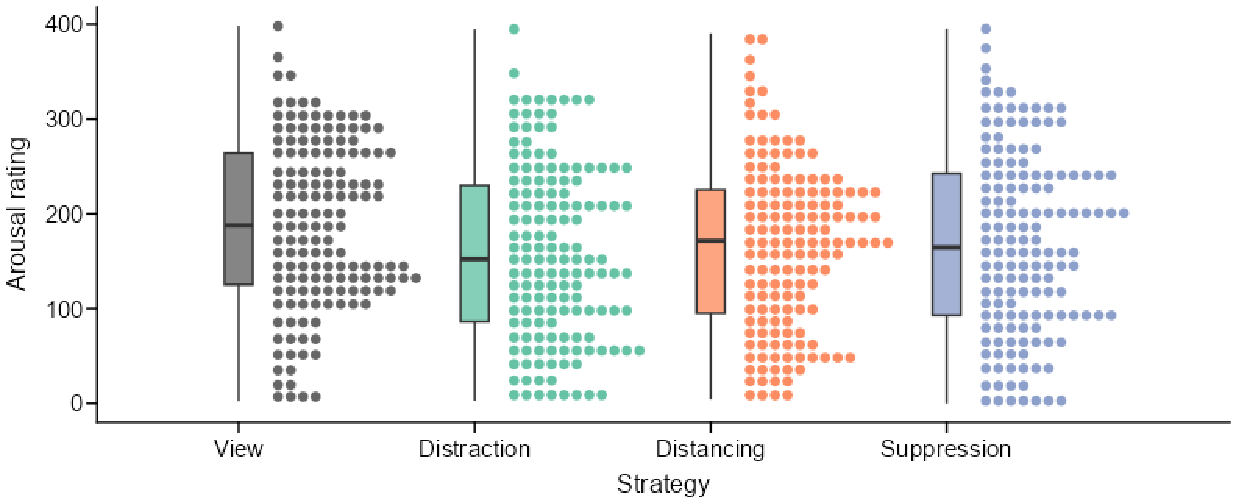

Figure S.4. Subjective arousal ratings visualized as boxplots. Dots represent individual effort ratings placed in 150 quantiles.

Table S.8

Post-hoc contrasts for effects of ER strategies on Corrugator activity

Contrast	Estimate	SE	df	t	p	$BF10$	η_p^2	$95\%CI$
$View_{neg} - Distraction$	0.178	0.037	351.000	4.788	0.000	21,919.73	0.06	[0.03, 1.00]
$View_{neg} - Distancing$	0.189	0.037	351.000	5.091	0.000	139,814.01	0.07	[0.03, 1.00]
$View_{neg} - Suppression$	0.210	0.037	351.000	5.669	0.000	1.84×10^{7}	0.08	[0.04, 1.00]
$Distraction - Distancing$	0.011	0.037	351.000	0.303	1.000	3.77×10^{-2}	2.61e-04	[0.00, 1.00]
$Distraction - Suppression$	0.033	0.037	351.000	0.881	1.000	8.02×10^{-2}	2.21e-03	[0.00, 1.00]
$Distancing - Suppression$	0.021	0.037	351.000	0.578	1.000	4.79×10^{-2}	9.51e-04	[0.00, 1.00]

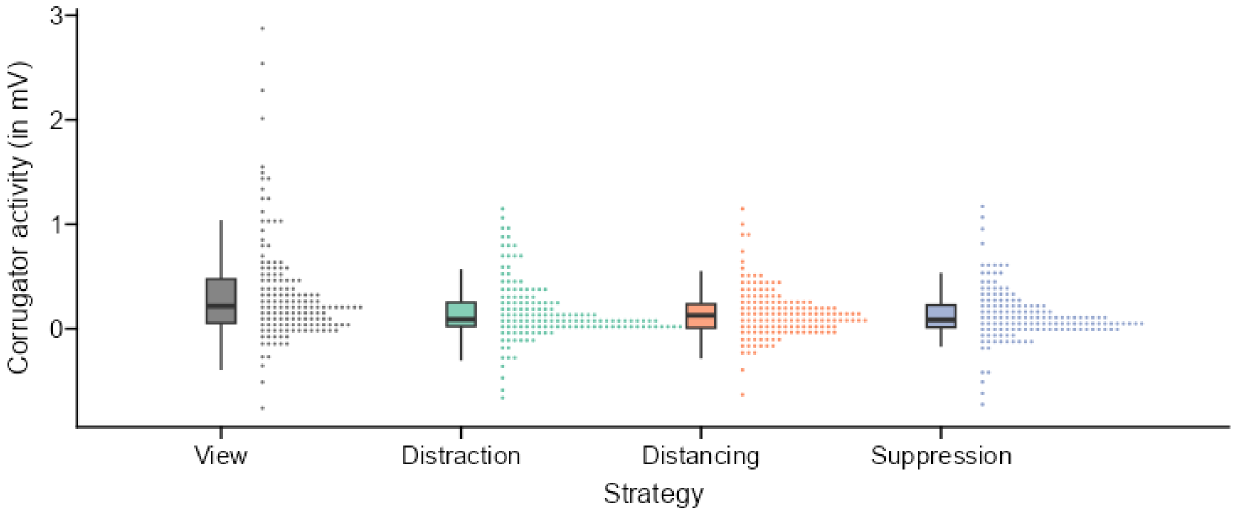

Figure S.5. Corrugator activity in mV visualized as boxplots. Dots represent individual Levatorr activity measures placed in 150 quantiles.

Table S.9

Post-hoc contrasts for effects of ER strategies on Levator activity

Contrast	Estimate	SE	df	t	p	$BF10$	η_p^2	$95\%CI$
$View_{neg} - Distraction$	0.336	0.050	351.000	6.731	0.000	2.02×10^{11}	0.11	[0.07, 1.00]
$View_{neg} - Distancing$	0.282	0.050	351.000	5.659	0.000	3.99×10^{7}	0.08	[0.04, 1.00]
$View_{neg} - Suppression$	0.318	0.050	351.000	6.370	0.000	8.60×10^{10}	0.10	[0.06, 1.00]
$Distraction - Distancing$	-0.053	0.050	351.000	-1.072	1.000	0.22	3.26e-03	[0.00, 1.00]
$Distraction - Suppression$	-0.018	0.050	351.000	-0.361	1.000	3.91×10^{-2}	3.70e-04	[0.00, 1.00]
$Distancing - Suppression$	0.035	0.050	351.000	0.711	1.000	9.86×10^{-2}	1.44e-03	[0.00, 1.00]

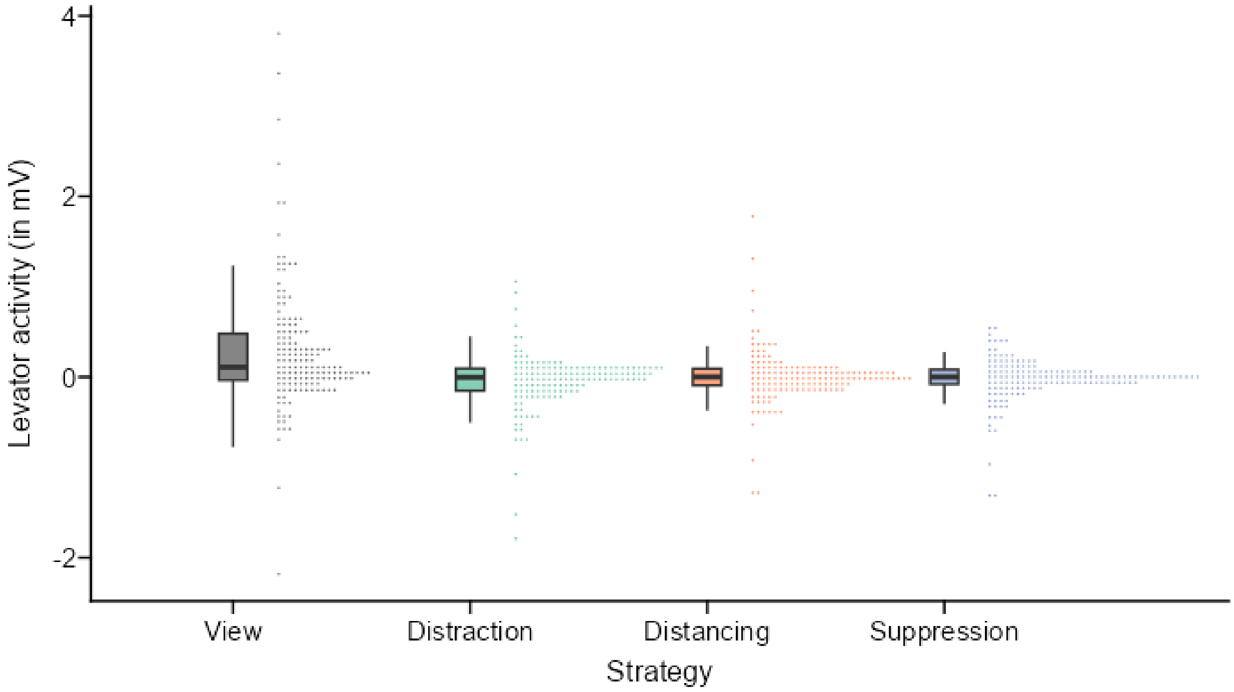

Figure S.6. Levator activity in mV visualized as boxplots. Dots represent individual Levator activity measures placed in 150 quantiles.

Exploratory analysis: Association between SVs and self-control and NFC

Table S.10

Exploratory analysis: Results of MLM predicting SVs of ER strategies with level 2 predictors self-control and NFC.

Parameter	Beta	SE	p-value	f^2	Random Effects (SD)
Intercept	8.03×10^{-1}	0.011	0.000		0.112
Effort	-6.93×10^{-4}	0.000	0.000	0.036	
Utility	1.44×10^{-3}	0.000	0.000	0.197	
Corrugator activity	7.54×10^{-3}	0.004	0.034	0.001	
Self-Control	2.44×10^{-2}	0.012	0.044	0.002	
NFC	7.58×10^{-4}	0.001	0.436	0.002	

1. Bech, P. Measuring the dimensions of psychological general well-being by the WHO-5. *Quality of life newsletter* **32**, 15–16 (2004).

2. Brähler, E., Mühlan, H., Albani, C. & Schmidt, S. Teststatistische prüfung und normierung der deutschen versionen des EUROHIS-QOL lebensqualität-index und des WHO-5 wohlbefindens-index. *Diagnostica* **53**, 83–96 (2007).

3. Connor, K. M. & Davidson, J. R. Development of a new resilience scale: The connor-davidson resilience scale (CD-RISC). *Depression and Anxiety* **18**, 76–82 (2003).

4. Campbell-Sills, L. & Stein, M. B. Psychometric analysis and refinement of the connor-davidson resilience scale (CD-RISC): Validation of a 10-item measure of resilience. *Journal of Traumatic Stress* **20**, 1019–28 (2007).

5. Sarubin, N. *et al.* First analysis of the 10-and 25-item german version of the connor-davidson resilience scale (CD-RISC) regarding psychometric properties and components. *Zeitschrift Fur Gesundheitspsychologie* **23**, 112–122 (2015).

6. Gross, J. J. & John, O. P. Individual differences in two emotion regulation processes: Implications for affect, relationships, and well-being. *Journal of Personality and Social Psychology* **85**, 348–62 (2003).

7. Abler, B. & Kessler, H. Emotion regulation questionnaire - a german version of the ERQ by gross and john. *Diagnostica* **55**, 144–152 (2009).

8. Preece, D. A., Becerra, R., Robinson, K. & Gross, J. J. The emotion regulation questionnaire: Psychometric properties in general community samples. *J Pers Assess* **102**, 348–356 (2020).

9. Dörfel, D., Gärtner, A. & Strobel, A. A new self-report instrument for measuring emotion regulation flexibility. *Society for Affective Science (SAS) Annual Conference* (2019).

10. Bernecker, K. & Job, V. Implicit theories about willpower in resisting temptations and emotion control. *Zeitschrift Fur Psychologie-Journal of Psychology* **225**, 157–166 (2017).

11. Bless, H., Wanke, M., Bohner, G., Fellhauer, R. F. & Schwarz, N. Need for cognition - a scale measuring engagement and happiness in cognitive tasks. *Zeitschrift Für Sozialpsychologie* **25**, 147–154 (1994).

12. Fleischhauer, M. *et al.* Same or different? Clarifying the relationship of need for cognition to personality and intelligence. *Personality & Social Psychology Bulletin* **36**, 82–96 (2010).

13. Fleischhauer, M., Strobel, A. & Strobel, A. Directly and indirectly assessed Need for Cognition differentially predict spontaneous and reflective information processing behavior. *Journal of Individual Differences* **36**, 101–109 (2015).

14. Schwarzer, R., Diehl, M. & Schmitz, G. S. Self-regulation scale. (1999).

15. Tangney, J. P., Baumeister, R. F. & Boone, A. L. High self-control predicts good adjustment, less pathology, better grades, and interpersonal success. *Journal of Personality* **72**, 271–324 (2004).

16. Sproesser, G., Strohbach, S., Schupp, H. & Renner, B. Candy or apple? How self-control resources and motives impact dietary healthiness in women. *Appetite* **56**, 784–787 (2011).

17. Patton, J. H., Stanford, M. S. & Barratt, E. S. Factor structure of the barratt impulsiveness scale. *Journal of Clinical Psychology* **51**, 768–774 (1995).

18. Hartmann, A. S., Rief, W. & Hilbert, A. Psychometric properties of the german version of the barratt impulsiveness scale, version 11 (BIS-11) for adolescents. *Perceptual and Motor Skills* **112**, 353–368 (2011).

19. Derryberry, D. & Reed, M. A. Anxiety-related attentional biases and their regulation by attentional control. *Journal of abnormal psychology* **111**, 225–236 (2002).
